# Vegetative Insecticidal Protein (Vip): A Potential Contender From *Bacillus thuringiensis* for Efficient Management of Various Detrimental Agricultural Pests

**DOI:** 10.3389/fmicb.2021.659736

**Published:** 2021-05-13

**Authors:** Mamta Gupta, Harish Kumar, Sarvjeet Kaur

**Affiliations:** ^1^ICAR-National Institute for Plant Biotechnology, New Delhi, India; ^2^ICAR-Indian Institute of Maize Research, Ludhiana, India; ^3^Punjab Agricultural University, Regional Research Station, Faridkot, India

**Keywords:** *Bacillus thuringiensis*, *vip* genes, Vip proteins, toxicity, resistance

## Abstract

*Bacillus thuringiensis* (*Bt*) bacterium is found in various ecological habitats, and has natural entomo-pesticidal properties, due to the production of crystalline and soluble proteins during different growth phases. In addition to Cry and Cyt proteins, this bacterium also produces Vegetative insecticidal protein (Vip) during its vegetative growth phase, which is considered an excellent toxic candidate because of the difference in sequence homology and receptor sites from Cry proteins. Vip proteins are referred as second-generation insecticidal proteins, which can be used either alone or in complementarity with Cry proteins for the management of various detrimental pests. Among these Vip proteins, Vip1 and Vip2 act as binary toxins and have toxicity toward pests belonging to Hemiptera and Coleoptera orders, whereas the most important Vip3 proteins have insecticidal activity against Lepidopteran pests. These Vip3 proteins are similar to Cry proteins in terms of toxicity potential against susceptible insects. They are reported to be toxic toward pests, which can’t be controlled with Cry proteins. The Vip3 proteins have been successfully pyramided along with Cry proteins in transgenic rice, corn, and cotton to combat resistant pest populations. This review provides detailed information about the history and importance of Vip proteins, their types, structure, newly identified specific receptors, and action mechanism of this specific class of proteins. Various studies conducted on Vip proteins all over the world and the current status have been discussed. This review will give insights into the significance of Vip proteins as alternative promising candidate toxic proteins from *Bt* for the management of pests in most sustainable manner.

## Introduction

*Bacillus thuringiensis* (*Bt*) is an aerobic, gram-positive, entomopathogenic bacterium. It is indigenous to various environments having most advantageous growth temperature at around 25–35°C (Martin and Travers, 1989; Bernhard et al., 1997). The various strains of this bacterium have been isolated globally from the diverse ecosystems such as soil (DeLucca et al., 1979, DeLucca et al., 1982; Dulmage and Aizawa, 1982; Martin and Travers, 1989; Carozzi et al., 1991; Hastowo et al., 1992; Bernhard et al., 1997; Hossain et al., 1997; Bravo et al., 1998), stored grain dusts (Meadows et al., 1992; Kaelin et al., 1994), foods (Damgaard et al., 1996; Rosenquist et al., 2005; Frederiksen et al., 2006), mills (Meadows et al., 1992), freshwater (Espinasse et al., 2003), insect cadavers (Chilcott and Wigley, 1993; Apoyolo et al., 1995; Damgaard et al., 1997; Hansen et al., 1998; Eilenberg et al., 2000), crustaceans (Swiecicka et al., 2006), annelids (Hendriksen and Hansen, 2002), marine sediments (Maeda et al., 2000) and insectivorous mammals (Świȩcicka and De Vos, 2003), rhizosphere (Manonmani et al., 1991; Hendriksen and Hansen, 2002; Bisht and Mishra, 2013) and phylloplanes (Smith and Couche, 1991; Damgaard et al., 1998; Hansen et al., 1998; Kaur and Singh, 2000, Hendriksen and Hansen, 2002; Collier et al., 2005; Bizzarri and Bishop, 2007). Therefore, with an uncertain ecology, this ubiquitous bacterium could be best explained as an opportunist pathogen. Despite the complex ecology of *Bt* strains, it has been proposed that this bacterium generally uses insect cadavers for reproduction (Raymond et al., 2010), which indicates the transient existence of *Bt* strains in these environments.

The genome size of *Bt* strains ranges from 2400 to 5700 kb. Its genome consists of chromosomal and 1 to >12 extrachromosomal elements (Carlson et al., 1994, 1996). Plasmid size in strains varies from 4.56 to 228 kb (Lereclus et al., 1982; Kolstø et al., 2009; He et al., 2011). This bacterium forms oval shape endospores, which do not inflate the sporangium and produce lecithinase as the preferred energy source. *Bt* is identified by the presence of one or more parasporal bodies (recognized as the crystal), which are observable within the sporangia utilizing a phase-contrast microscope (Schnepf et al., 1998). The life cycle of *Bt* consists of two stages, *i.e*., vegetative and sporulation stages. Cell division is carried out in the vegetative stage, whereas spore formation occurs in the sporulation phase (Bechtel and Bulla, 1982; Lambert and Peferoen, 1992).

*Bacillus thuringiensis-*based biopesticides have become a vital part of insect pest management strategies. This bacterium has successfully been utilized as a source of *cry* genes for plant genetic engineering to develop transgenic crops showing resistance toward various detrimental insect pests (Tohidfar and Salehi Jouzani, 2008; Tohidfar et al., 2013; Melo et al., 2016; Tabashnik and Carrière, 2017). *Bt* has also, the capability to be utilized as a nematicide because it synthesizes metalloproteinase, thuringiensin, and chitinase, which are toxic to plant pathogenic nematodes (Jouzani et al., 2008; de la Fuente-Salcido et al., 2013; Iatsenko et al., 2014). In addition to Cry and Cyt toxins, *Bt* strains can also produce other insecticidal proteins during the vegetative growth phase. These toxins are consequently secreted into the culture medium and have been named vegetative insecticidal proteins (Vip) (Estruch et al., 1996; Warren et al., 1998) and the secreted insecticidal protein (Sip) (Donovan et al., 2006).

## Vegetative Insecticidal Proteins (Vips)

Many researchers demonstrated that *Bt* strains secreted Vip (Vegetative insecticidal protein) proteins during the vegetative growth phase and these proteins do not share any structural and sequence homology with Cry proteins, therefore these are considered as a tremendous complement or supplement source of Cry toxins in resistance management and crop protection. Various commercial *Bt*-crops combine Vip3 and Cry proteins. The approach of pyramiding of toxic proteins having different modes of action has been gaining importance as a viable strategy for the management of development of resistance in target insects (Hernández-Rodríguez et al., 2009). Seven *cry* and *vip* genes, namely *cry1Ab*, *cry2Ab*, *cry1Ac*, *vip3A*, *cry1F*, and *cry2Ae* have been utilized to improve resistance against lepidopteran pests. Gene-pyramiding systems, in which *Bt* crops have more than *cry* or *vip* genes, have been developed to reduce the probability of development of the resistant population of insect pests against *Bt* toxins produced in transgenic crops (Jouzani et al., 2017).

### Types, Host Specificity, and Characteristics

**FIGURE 1:**
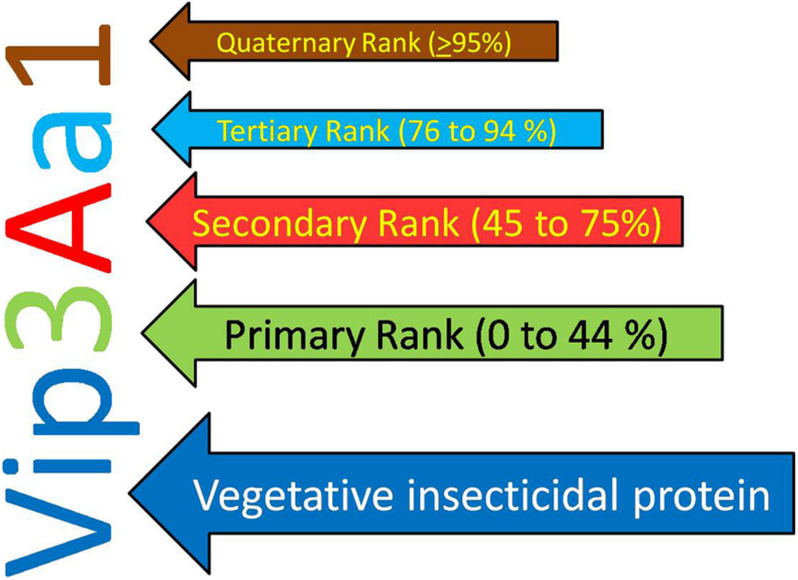
New nomenclature criteria for Vegetative Insecticidal Protein given by nomenclature committee.

The earlier nomenclature of the Vip proteins includes four ranks based on the identity of the amino acid sequence. The primary rank differentiates proteins with <45% sequence identities, whereas secondary and tertiary ranks differentiate proteins with up to 78 and 95% sequence identities, respectively. Further proteins sharing >95% sequence identity is given quaternary rank, which might be described as “allelic” forms of the similar gene but may also contain the similar sequence that originated from other isolates. Such as, Vip1 and Vip2, if they have <45% sequence identity, Vip3A and Vip3C if they have <78% sequence identity, Vip3Aa and Vip3Ab if they have <95% sequence identity, and Vip3Aa1 and Vip3Aa2 if they sequence >95% sequence identity (Crickmore et al., 1998). Recently, a new nomenclature criterion for bacterial pesticidal proteins has been published. According to this nomenclature system, primary rank represents the proteins having 0 to 44% sequence identity; secondary rank and tertiary rank represents the proteins having 45–75 and 76–94% sequence identity, respectively, and the quaternary rank determined based on >95% identity (Crickmore et al., 2020; Figure 1).

#### Old classification of Vip Proteins: Based on Sequence Homology

According to the old classification system, 147 diverse Vip proteins have been identified and classified into four families Vip1, Vip2, Vip3, and Vip4 based on their sequence homology (^1^ accessed on 15.01.2021). Among these Vip proteins, Vip1 and Vip2 proteins are collectively known as binary toxins of A-B type and their combination have an insecticidal effect against Coleopteran and Hemipteran pests (Warren, 1997; Sattar and Maiti, 2011). Among the family of Vip toxic proteins, Vip3A proteins are most studied, present in the highest numbers, and toxic against insects belonging to the Lepidoptera order (Palma et al., 2014; Chakroun et al., 2016). Till now, 111 Vip3 proteins have been isolated from *Bt* strains and isolates and classified into three families named Vip3A, Vip3B, and Vip3C. Based on DNA-sequence similarity, these families are grouped into 10 Vip3A sub-families (Vip3Aa to Vip3Aaj), three Vip3B sub-families (Vip3Ba to Vip3Bc), and one Vip3C sub-family (Vip3Ca). There are total 101 Vip3A-type proteins are reported until now and out of these, sixty seven Vip3Aa (Vip3Aa1 to Vip3Aa66 and Vip3Aa19.0), two Vip3Ab (Vip3Ab1 and Vip3Ab2), one Vip3Ac (Vip3Ac1), six Vip3Ad (Vip3Ad1 to Vip3Ad6), one Vip3Ae (Vip3Ae1), four Vip3Af (Vip3Af1 to Vip3Af4), fifteen Vip3Ag (Vip3Ag1 to Vip3Ag15), two Vip3Ah (Vip3Ah1 and Vip3Ah2) one Vip3Ai (Vip3Ai1), two Vip3Aj (Vip3Aj1 and Vip3Aj2) are available in Vip toxin database of *Bt*. In addition to this, two Vip3Ba (Vip3Ba1 and Vip3Ba2), three Vip3Bb (Vip3Bb1, Vip3Bb2, and Vip3Bb3), one Vip3Bc and four Vip3Ca (Vip3Ca1 to Vip3Ca4) are also available in the database (see text footnote 1 accessed on 15.01.2021). A novel protein, designated as Vip4Aa1, has been discovered in 2010 (Palma et al., 2014).

#### New Classification of Vip Proteins: Based on Structural Homology

**FIGURE 2:**
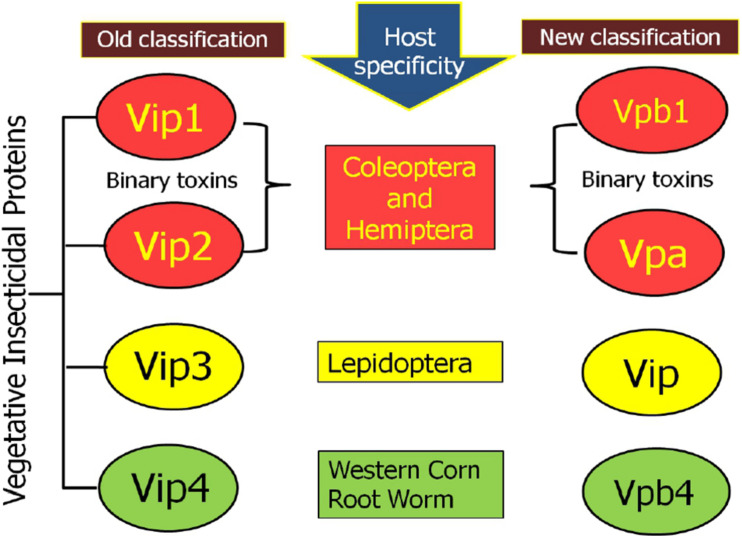
Schematic representation of comparative changes in the old and new classification system for Vip family protein.

**FIGURE 3:**
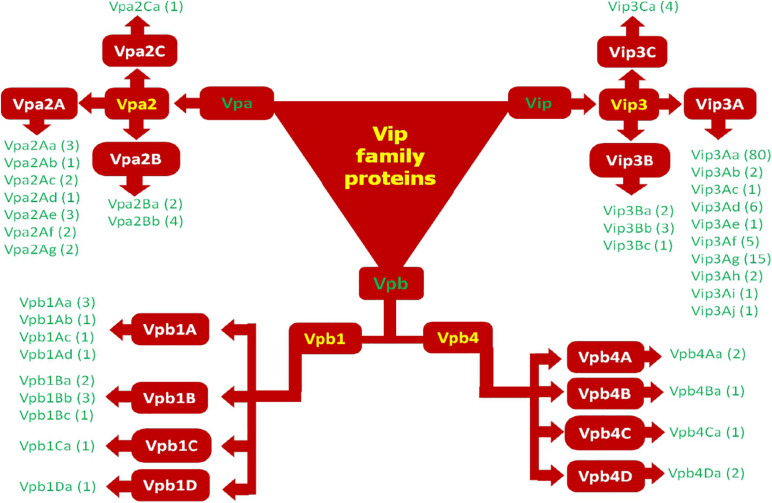
Modern classification of Vip family proteins (Vip1 as Vpb1; Vip2 as Vpa2; Vip3 as Vip3; and Vip4 as Vpb4).

According to the new classification system, Vip1 and Vip4 have been renamed as Vpb1 and Vpb4, respectively, representing Vpb group, due to their structural similarity. Previously known catalytic Vip2 protein has been categorized as Vpa group of pesticidal protein. Finally, Vip3 proteins having a multi-domain structure have been noted as Vip (Vegetative insecticidal proteins) in the new classification of bacterial pesticidal proteins, and all proteins belonging to this class retain their original name from old nomenclature. Thus, previously classified 4 types of Vip proteins have been categorized into three different classes as Vpa, Vpb, and Vip (Crickmore et al., 2020^2^). Thus, to date, 21 Vpa2 proteins (^3^ accessed on 15.01.2021), including 14 Vpa2A (Vpa2Aa1 to Vpa2Aa3; Vpa2Ab1; Vpa2Ac1 to Vpa2Ac2; Vpa2Ad1; Vpa2Ae1 to Vpa2Ae3; Vpa2Af1 to Vpa2Af12 and Vpa2Ag1 to Vpa2Ag2); 6 Vpa2B (2 Vpa2Ba1 to Vpa2Bb2; Vpa2Bb1 to Vpa2Bb4) and one Vpa2C (Vpa2Ca1) have been reported. The Vpb proteins have been classified into 14 Vpb1 and 6 Vpb4 proteins (^4^ accessed on 15.01.2021). The Vpb1 group have total 14 proteins, including six Vpb1A (Vpb1Aa1 to Vpb1Aa3; Vpb1Ab1; Vpb1Ac1 and Vpb1Ad1); six Vpb1B (Vpb1Ba1 to VpbBa2; Vpb1Bb1 to Vpb1Bb3 and Vpb1Bc1), one Vpb1C (Vpb1Ca1) and one Vpb1D (Vpb1Da1). The Vpb4 have 6 proteins categorized as Vpb4A (Vpb4Aa1 to Vpb4Aa2); Vpb4B (Vpb4Ba1); Vpb4C (Vpb4Ca1) and Vpb4D (Vpb4Da1 to Vpb4Da2). A total of 124 Vip3 proteins are available in new database of pesticidal proteins (^5^ accessed on 15.01.2021) including 114 Vip3A (Vip3Aa1 to Vip3Aa80; Vip3Ab1 to Vip3Ab2; Vip3Ac1; Vip3Ad1 to Vip3Ad6; Vip3Ae1; Vip3Af1 to Vip3Af5; Vip3Ag1 to Vip3Ag15; Vip3Ah1 to Vip3Ah2; Vip3Ai1; Vip3Aj1 to Vip3Aj2); 6 Vip3B (Vip3Ba1 to Vip3Ba2; Vip3Bb1 to Vip3Bb3 and Vip3Bc1) and 4 Vip3C (Vip3Ca1 to Vip3Ca4). The new classification system of Vip family proteins and their grouping has been depicted in Figures 2, 3. The characteristics of each protein are mentioned in Table 1.

**TABLE 1:** Characteristics of proteins belonging to VIP family.

Old/New Name*	Vip1/Vpb1	Vip2/Vpa2	Vip3/Vip3	Vip4/Vpb4
Total number in database	14	21	110	6
Gene Length	4 to 5 kb separated by 4–16 bp intergenic spacer		∼2.4 kb	∼2.9 kb
Signal peptide (aa)	35	50	12–188	1–28
Protoxin Protein size (kDa)	100	52	89	∼ 108
Active Protein size (kDa)	82	45	62–66 along with N-terminus 19 kDa	NA
Amino Acids (aa)	881	462	787–789	937–965
Domain	Binary_ToxB exotoxin bacterial domain	(1) N-terminal (60–265 aa) (2) NAD-binding domain (266–461 aa)	(1) Domain I (1–199 aa): essential for activity; (2) Domain II (200–325): Stabilization of oligomer; Domain III (328–536 aa): interacts with domain I; Domain IV and V: Carbohydrate binding motifs	**Vpb4Aa1** (1) PA14 domain, (2) BinaryTox B domain **Vpb4Da2** (1) PA14 domain; (2) Binary_Tox B (3) Binary ToxB_2 and (4) Binary Tox_3 domains
Conserved domain name	pfam07691, pfam03495, pfam17475, pfam17476	cd00233	pfam12495, pfam02018	pfam07691, pfam03495, pfam17475, pfam17476
PDB code	6SMS	1QS2	6V1V	6SMS
Mode of Action	A+ B type binary toxins: B (Vip1/Vpb1) as Receptor binding domain and A Vip2/Vpa2) as Cytotoxic domain with ADP-ribosyltransferase activity and inhibiting polymerization of actin filaments (Disintegration of insect cytoskeletion)		Apoptosis/Pore formation	NA
Target pest/Insect order	Hemipteran and Coleopteran		Lepidopteran	Vpb4Da2 against Western corn root worm (*Diabrotica virgifera virgifera* LeConte) (Yin et al., 2020)
Transgenic Crop	NA		Maize and Cotton (Commercially available)	Maize
References	Warren et al. (1998); Shi et al. (2007); Sattar and Maiti (2011); Bi et al. (2015); Chakroun et al. (2016); Crickmore et al. (2020)		Estruch et al. (1996); Lee et al. (2003); Rang et al. (2005); Ruiz de Escudero et al., 2014; Zack et al. (2017); Chakrabarty et al. (2020); Crickmore et al. (2020); Núñez-Ramírez et al. (2020)	Palma et al. (2014); Chakroun et al. (2016); Yin et al. (2020); Crickmore et al. (2020)

*As per new nomenclature assigned by *Bt* nomenclature committee and available as pesticidal protein database (www.bpprc.org).

### Vip1 and Vip2

#### Structure of Binary Toxin Vip1 and Vip2

In *Bacillus* species, the genes *vip1A* and *vip2A* are positioned in one operon and encode 100 and 52 kDa proteins, respectively (Warren et al., 1998; de Maagd et al., 2003). The binary toxin has two components *viz.* A and B. “A” component includes Vip2 protein whereas “B” component involves Vip1 protein (Barth et al., 2004). Vip1 is a channel-forming protein, which works as the binding and translocation component (Blaustein et al., 1989; Schmid et al., 1994; Knapp et al., 2002) and Vip2 goes into the cell and cause toxicity. Sequence analysis reveals that Vip2 has two distinguish domains: the N-terminal domain (60 to 265 amino acids), whereas the C-terminal domain (of 266 to 461 amino acids), a NAD-binding domain. Although both domains of Vip2 share very less sequence homology but crystallography structure analysis reveals that they have structural homology to each other. The core of each domain is mostly created by the vertical packing of a five-stranded mixed β-sheet with a three-stranded antiparallel β-sheet. Four consecutive α-helices flank the three-stranded β-sheet, whereas an additional α-helix flanks the five-stranded β-sheet (Han et al., 1999). Further, N-terminal signal peptides are cleaved during secretion and the mature proteins of 45 kDa (for Vip2Aa) and 82 kDa (for Vip1Aa) are delivered (Warren, 1997; Bi et al., 2015). Sequence alignment studies showed that with 75 to 91% identity, the N terminus of Vip1 is considered to be highly conserved, whereas the C terminus of Vip1 is less conserved with 23 to 35% identity (Warren, 1997; Shi et al., 2004; Shi et al., 2007). Conclusively, Vip1 and Vip2 proteins were constituted with domains of receptor binding and cytotoxic domain, respectively (de Maagd et al., 2003). Simultaneously, another study reported that after purification from *Bt*#BREF24 isolate, the aphidicidal protein was recognized as a binary toxin. *Bt*#BREF24 was used to clone the binary toxin of the *vip2Ae* and *vip1Ae* genes. Both proteins components of the binary toxins were essential for the activity, as confirmed by the Aphid feeding assay. Through ligand blotting experiment, they identified a ∼50 kDa receptor in the brush border membrane vesicles of the cotton aphids only, but not in the lepidopteran insects. So, these proteins can be used against sap-sucking insect pests (Sattar and Maiti, 2011). Osman et al. (2013) identified *vip1* (2.3 kb) and *vip2* (1.3 kb) bands in only 4 strains out of 100, which were cloned, sequenced and analyzed.

#### Vip1/Vip2 Mode of Action

Vip1 and Vip2, the coleopteran and homopteran-specific toxins, respectively, work in binary action as “A-B type” (Warren et al., 1998; de Maagd et al., 2003; Yu et al., 2011a). The receptor-binding domain of Vip1 binds with the midgut membrane receptors of the target insect as monomer or oligomer and forms the pore/channel through which the cytotoxic domain of Vip2 protein exerts its action through ADP-ribosyl transferase in the cytoplasm of insect. Vip2 can also enter the cell through endocytosis (Leuber et al., 2006; Pardo-Lopez et al., 2013; Chakroun et al., 2016). The selectivity of channel formation in Vip1 is supposed to be regulated by the presence of negatively charged glutamic acid (position 340 and 345), and positively charged Lysine residues (351), and Histidine (363) (Leuber et al., 2006). There are two hypotheses regarding the entry of Vip2 into target cells. First, the homology of Vip2 with binary toxin C2 clostridial (I component) supports its entry through endocytosis (Barth et al., 2004). The second hypothesis is the presence of powerful proton gradient force sustained by the midgut’s alkaline solution, which directly enters the cytoplasm of the target cell through a pore formed by Vip1 (Leuber et al., 2006). After entering the cytoplasm, Vip2 protein acts against actin in targets cells by preventing the formation of microfilaments through ADP-ribose catalytic transfer from NAD to actin and disintegration of insect’s cytoskeleton, resulting in the death of target insect (Han et al., 1999; Jucovic et al., 2008; Aktories et al., 2011).

### Vip3 Proteins

Vip3 proteins are reported to be effective against black cutworm *Agrotis ipsilon* (Yu et al., 1997; Donovan et al., 2001), cotton boll worm; *Helicoverpa armigera* (Lee et al., 2003; Cai et al., 2006; Fang et al., 2007), Beet army worm *Spodoptera exigua* (Donovan et al., 2001; Cai et al., 2006), fall armyworm *S. frugiperda* (Yu et al., 1997; Sena et al., 2009; Figueiredo et al., 2013), tobacco budworm *Heliothis virescens* (Estruch et al., 1996; Gulzar and Wright, 2015), tobacco hornworm *Manduca sexta* (Lee et al., 2003), African cotton leafworm *S. littoralis*, European grapevine moth *Lobesia botrana*, Cabbage moth *Mamestra brassicae* (Palma et al., 2013; De Escudero et al., 2014), Mediterranean flour moth *(Ephestia kuehniella*) (Mesrati et al., 2005), cabbage looper *Trichoplusia ni* (Fang et al., 2007), Tomato leaf miner *Tuta absoluta* (Sellami et al., 2015), diamondback moth *Plutella xylostella* (Bhalla et al., 2005; Palma et al., 2013; Gulzar and Wright, 2015), locust bean moth *Ectomyelois ceratoniae* (Boukedi et al., 2015) and tobacco cutworm *S. litura* (Bhalla et al., 2005; Song et al., 2016).

*Agrotis ipsilon* is a significant agricultural pest, which shows some tolerance against delta-endotoxins. However, Vip3A caused 100% mortality of *A. ipsilon* (Estruch et al., 1996) and showed toxic activity against this pest 260-times higher than Cry1A proteins (Yu et al., 1997). Lee et al. (2003) reported no insecticidal activity against the non-target beneficial insect *Danaus plexippus* L. (Monarch butterfly). Rang et al. (2005) found that Vip3Ba1 does not show larvicidal effect but delays the growth of *O. nubilalis* and *P. xylostella*. Fang et al. (2007) showed that Vip3Ac1 was toxic against *S. frugiperda*, whereas both Vip3Aa1 and Vip3Ac1 were not toxic against *O. nubilalis*. Further, Vip3AcAa, a chimeric protein, constructed by combining both Vip3Aa1 and Vip3Ac1, became toxic to *O. nubilalis* and showed enhanced toxicity against *S. frugiperda*. These authors also demonstrated that resistance to Cry1A does not confer cross-resistance to Vip3A because cabbage looper (Lepidoptera: Noctuidae) showed resistance toward Cry1Ac but was susceptible to Vip3Ac1 and Vip3AcAa.

#### Vip 3 Proteins: Structure

Various studies have been done to focus on the protein structure of this class. It was suggested that helices formed the N-terminal and the C-terminal was composed of helices and coil structures (Rang et al., 2005; Wu et al., 2007). It was reported that deletions in N and C-terminal of Vip3 proteins revealed a different effect on toxicity as deletion of 39 aa in N-terminal of Vip3 protein results in a notable reduction in toxicity against *S. litura* larvae, but the same deletion did not affect the toxicity against *C. partellus* larvae. Additionally, deletion of 154 aa in the C-terminal affected the toxicity against larvae of both insects in a differential manner. Their results suggested the possibility of differential action mechanism of Vip3 proteins concerning different target pests (Selvapandiyan et al., 2001). Contrasting to previous studies, Ndv200 (N-terminal deletion mutant of Vip3BR toxin) showed enhanced toxicity against *H. armigera*, *A. ipsilon*, *S. littoralis*, and *Scirpophaga incertulas* (Gayen et al., 2015).

Initially, it was demonstrated that deletion of 198 aa at N-terminal of Vip3Aa resulted in the entire loss of its toxic nature and produced trypsin sensitive 62 kDa protein (Li et al., 2007). In solution, the Vip3A (protoxin and activated form) protein exists as a homo-tetramer, and domain I was found to be essential for its formation. It was suggested that along with the active core of 65 kDa, the 19 kDa fragment is also essential for Vip3 insecticidal nature (Zack et al., 2017), and a low-resolution 3D structure of its tetrameric form has been elucidated (Kunthic et al., 2017; Palma et al., 2017). The secondary structure predicted for Vip3Aa protein expressed from Vip3Aa16 showed that the C-terminal region was composed of protease-resistant β-sheets clusters and the rest of the protein was made up with α-helices (Bel et al., 2017). The 3D structure of Vip3Af1 protein containing 5 domains was also revealed and it was confirmed that 19 amino acids are critical for insecticidal activity of this protein, as their substitution greatly reduced the toxicity of this protein against *Agrotis segetum* and *Spodoptera frugiperda*. These substitutions were mainly clustered into two regions 167–272 and 689–741 and two positions (483 and 552) in the middle of the gene. The critical amino acid substitutions in the region 167–272 were found at positions 167, 168, 171, 209, 229, 238, 242, 244, 246, 255, 272 and in the region 689–741 at positions 689, 699, 711, 719, 727, 741 (Banyuls et al., 2018). Subsequently, the three domains of Vip3Aa16 protein were predicted through *in silico* modeling similar to activated Cry proteins as (1) Domain I: N-terminal having hemolysin fold (7-bundle helix) and Domain II and III: CBM (Carbohydrate binding domain) which are specifically involved in receptor binding (Sellami et al., 2018).

Quan and Ferré (2019) identified 5 domains in Vip3Af protein structure by trypsin digestion and stability analysis: Domain I: 12–198 aa; Domain II: 199–313 aa; Domain III: 314–526; Domain IV: 527–668 aa; Domain V: 669–788 aa. It was demonstrated that tetrameric structure was formed by domain I, II, and III; not by domain V; while the function of domain IV was not clear. But the domain IV consists of CBM (carbohydrate-binding motif), which is common in all kinds of Vip3 proteins except Vip3Ba (Chakroun et al., 2016). Subsequently, Wang et al. (2019) created two engineered mutations in domain I (175 S/C and 177 L/C) and confirmed that Vip3A did not show toxic behavior against *Chilo suppressalis*. However, its ability to compete with binding sites of wild type remained intact. The study concluded that domain I and domain V were responsible for membrane insertion and binding specificity, respectively.

The first crystal structure of Vip3B2160 (60% identical to Vip3A proteins in terms of sequence) has been proposed by another group (Zheng et al., 2020). The conserved domain database of NCBI exhibited the presence of N-terminal Vip3A_N super family (pfam12495) between 12 and 188 and CBM_4_9 super family (pfam02018) from 536 to 654 amino acid residues. The N-terminal of Vip3A protein as a signal peptide might be involved in the secretion of protein, structural maintenance, and insecticidal activity, and the maximum variability found at C-terminal is responsible for target specificity (Zack et al., 2017; Chakrabarty et al., 2020). It was suggested that amino acid D199 to end (C-terminal core) represents the toxic core of Vip3Aa11 (Jiang et al., 2020).

The first crystal structure of the C-terminal fragment of Vip3A (Vip3Aa11) toxic has been elucidated by Jiang et al. (2020). The structural analysis demonstrated the presence of four and five domains in activated Vip3A and protoxin Vip3A, respectively. The five domains from N to C-terminal included Domain I (1–198 aa): Domain II (199–327 aa); Domain III (328–518 aa); and Domain IV (537–667 aa) and Domain V (679–789 aa).

According to the study of this crystal structure, the structure of activated Vip3Aa11 resembles in shape with that of lobster, with the body constituted of domain II and III and claws formed by domain IV and V. The compact relationship was observed between domain II and III while flexibility was seen among other domains.

**FIGURE 4:**
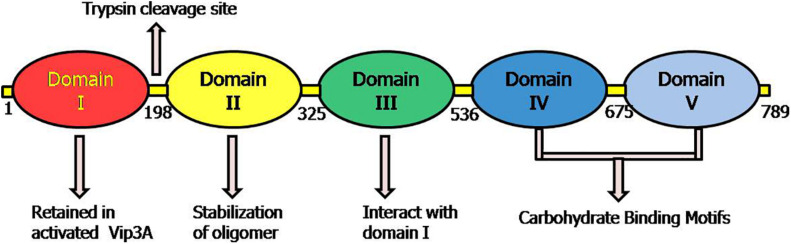
Representative picture of domains and their proposed role according to the 3D structure of Vip3 proteins as proposed by Núñez-Ramírez et al. (2020). The positions 1–789 depict the amino acid residues in Vip3A protein.

Recently, another new high-resolution 3D-structures of complete protoxin and activated Vip3Aa protein conformations were analyzed through Cryo-electron microscopy at 2.9 Å by Núñez-Ramírez et al. (2020). The domain organization of protoxin Vip3 revealed five domains in the protoxin (Figure 4), which are following:

Domain I (1–199aa): It is composed of highly curved four (α1–α4) helices that look like pyramid apex and validated as protease cleavage site. This study highlighted the importance of domain I (198 aa) at N-termini of Vip3 proteins as an essential portion of active toxin with supporting data and elucidated that after protease digestion, this domain is cleaved but always remains tightly associated with the core protein by interacting with domain II. This explained the reason behind the stability of Vip3 chimeras formed by exchanging this particular domain and instability of Vip3 mutants lacking domain I.

Domain II (200–325 aa): The five helices (α) of this domain represent the tetrameric core and the two extended loops (221–226 aa; 239–247 aa) have a structural role in the stabilization of Vip3 oligomer by heading toward nearby subunits.

Domain III (328–536 aa): The three antiparallel β-sheets of the third domain form the β-prism fold, but a precise role is yet to be validated. This domain has interactions with mostly segment (14–23 aa) of protein at the N-terminal.

Domain IV and V: Both domains are connected through a long linker and were found to be composed of carbohydrate-binding motifs (CBM). It was also elucidated that these domains have free contact with domain III due to their flexible nature.

Thus, this architecture revealed that tetramer of Vip3 protoxin assembled into pyramid shape having N-terminal apex and core, and C-terminal exposed toward solvents (Núñez-Ramírez et al., 2020). The structure-comparison analysis of Vip3A and Vip3B revealed that both proteins have similar tetrameric organization shares a significant similarity between domains I, II, and III and the analogous relationship between the flexible domains IV and V as they are responsible for exposing of glycan-binding side toward solvent (Núñez-Ramírez et al., 2020; Zheng et al., 2020).

##### Organization of domains in activated Vip3A

To determine the activation mechanism of purified protein after protease digestion, the 3D-structure of activated Vip3 was analyzed by Núñez-Ramírez et al. (2020) using electron microscopy. The results showed that near about 30 per cent of activated molecules retained the same configuration as in uncleaved protein. The remaining 70 per cent molecules formed the needle-like configuration. Thus, it was concluded that protease may be necessary for cleavage but there may be other factors also involved in the Vip3 activation. This activated toxin is also configured in a tetrameric conformation, in which, around the axis, four identical monomers are arranged along with tightly linked cleaved fragment. The architecture of domain II-V was similar to protoxin-form, but domain-I reorganized into a long needle of ∼200 Å.

These crystal structures and proposed domain organization may be representative of all Vip3 proteins because of the high degree of sequence homology among more than 100 Vip3 proteins available in the bacterial pesticidal protein database (Jiang et al., 2020; Núñez-Ramírez et al., 2020; Zheng et al., 2020).

#### Vip3 Proteins: Mode of Action and Their Receptors

Various studies have been performed to elucidate steps regarding the mode of action of Vip3 type of proteins. The Vip3A proteins can be solubilized at pH 5–10, which allows their solubilization in the alkaline insect midgut. Initially, the solubilization of Vip3A proteins, their proteolytic cleavage, and binding in insect midgut was demonstrated and correlated with toxicity effects like Cry toxins *viz*. feeding inhibition, activity loss of gut, paralysis, and death of target insect. But, the onset of symptoms develops after 48–72 h after Vip3A toxin ingestion, while it develops within 24 hr after ingestion of Cry toxins (Yu et al., 1997).

The binding receptors of Vip3A proteins on BBMV of insect midgut are also dissimilar from the receptors of Cry1A. The ligand blot analysis in the midgut of *Manduca sexta* confirmed that Vip3Aa proteins interact with proteins of 80 kDa and 110 kDa, while Cry1Ab interacts with 120 kDa and 210 kDa proteins, representing APN and cadherin kind of receptors, respectively (Lee et al., 2003). Further, the different binding sites of Vip3A and Cry2Ab or Cry1Ac were demonstrated in lepidopteran pests *H. zea* and *H. virescens* (Lee et al., 2006). Other studies showed the binding of Vip3Aa proteins with 65 kDa protein and Cry1Ac with 210 kDa protein in the midgut of *Prays oleae* (Mesrati et al., 2009) and *A. segetum* (Hamadou-Charfi et al., 2013). The binding of activated Vip3A with 55 kDa and 100 kDa ligands has been also demonstrated in the Mediterranean flour moth (*Ephestia kuehniella*) by Mesrati et al. (2011).

The extensive damage was observed in the midgut of susceptible insects after ingestion of Vip3A kind of proteins, which includes symptoms like swelling of cell, cytoplasm vacuolization, leakage of cell content, lysis in epithelial cells, and cell disintegration. These results confirmed that midgut is the target binding site for these proteins (Chakroun and Ferré, 2014; Boukedi et al., 2015). The voltage clamp assay also confirmed the presence of the pore formation step followed by binding with midgut receptors. The pores formation by gut-juice activated Vip3A-G was able to disturb transmembrane potential, which suggested the role of pore formation as an important contribution in the insecticidal activity of these proteins (Lee et al., 2003). It was confirmed that while these proteins do not show homology with crystalline toxins, they exert virulence following same sequential steps similar to Cry protein, such as ingestion of toxin by an insect; proteases activation in midgut; receptor binding, and formation of the pore (Palma et al., 2014; Chakroun et al., 2016).

These Vip3A toxins comprise of protein of approximately 800 amino acid residues of 89 kDa size having N-terminal (conserved region) and C-terminal (variable region). Li et al. (2007) observed that midgut juice extract/trypsin cleaved the 89 kDa Vip3A complete toxin into 62 kDa toxin., which was proposed to be required for its insecticidal properties. The midgut juice hydrolyzed the protoxin of Vip3A into different polypeptide fragments *viz*. 22, 33, 45, and 62–66 kDa. The first 198 aa of N-terminal produces 22 kDa peptide and the rest amino acid residues form 66 kDa core, which upon hydrolysis was found to produce 33 and 45 kDa peptides. This 66 kDa toxin has been reported to bind with brush border membrane vesicles (BBMV) of susceptible insect’s midgut (Lee et al., 2003; Gayen et al., 2012; Chakroun and Ferré, 2014; Bel et al., 2017). The interaction of 62 kDa Vip3Aa10 toxin with BBMV of cotton boll worm was studied and it was found to be specific and not shared with toxin Cry1Ab. The variation in insecticidal activity against different insects may be in part explained by the difference in protoxin-hydrolysis rates in the insect midgut, since the difference in affinity for the membrane receptors must have a strong influence on the toxicity (Chakroun et al., 2012; Boukedi et al., 2015). Competitive binding experiments with *S. frugiperda* conducted by Chakroun and Ferré (2014), revealed that these proteins don’t share binding sites with Cry proteins (Cry1Ab, Cry1Ac, Cry1Fa, Cry 2Ab, and Cry2Ae). The main processes involved in the cytotoxic properties of Vip3A are proposed as the formation of pore and/ory apoptosis after target cell binding (Lee et al., 2003; Jiang et al., 2016; Hernández-Martínez et al., 2013). Various studies have demonstrated that both Cry and Vip3 toxins do not have common receptors in insect midgut, due to structural diversity (Mesrati et al., 2009; Sena et al., 2009; Gouffon et al., 2011), but they share the receptors within the same group, *i.e*., Vip3Aa, Vip3Ad, Vip3Ae, and Vip3Af or with another group like Vip3Ca (Chakroun and Ferré, 2014; Gomis-Cebolla et al., 2017). The interaction between Vip3A protein and S2-ribosomal protein present on the Sf21 cell line of *Spodoptera frugiperda* was validated through yeast two-hybrid system, *in vitro* pull-down assays, and knockdown of expression of S2 protein (RNA interference). The knockdown of S2 protein resulted in the reduction of Vip3 protein toxicity. The study with a confocal microscope revealed the interaction between Vip3 and S2 proteins in the cytoplasm and surface of these cells. This interaction results in lysis of Sf21 cells leading to insect death (Singh et al., 2010). Recently, three kinds of Vip3-receptors have been identified in different insects, as discussed below:

I. Tenascins-like Black cut worm Vip3Aa-Receptor

The binding of Vip3Aa protein with 48 kDa receptor, present in epithelial tissue of *A. ipsilon* midgut has been demonstrated. Its gene size was 1.37 kb and deduced protein sequence showed the homology with tenascins (belonging to extracellular glycoproteins). The role of the receptor has also been speculated to aggregate and be involved in the formation of channel during the transport of nutrients. It was hypothesized that the Vip3A proteins may cause interference in the function of receptors either channel formation or nutrient transport (Osman et al., 2019).

II. *Spodoptera frugiperda*-Fibroblast Growth factor Receptor (*Sf*-FGFR)

The mass spectrometry and magnetic bead fishing analysis revealed the binding of Vip3Aa protein with FGFR receptor present on Sf 9 cell lines of *S. frugiperda*. It was indicated that both proteins were internalized into Sf9 cells and knockdown of FGFR resulted in the development of resistance against Vip3Aa. Hence, *Sf*-FGFR is declared as a receptor of FGFR. This receptor is similar to the tyrosine kinase receptor subfamily. The kinase receptors are mainly involved in the physiological activities of cells. The FGFR1 receptor present in mammalian cells is mainly involved in the apoptosis of cancer cells in the lungs. Therefore, it has been concluded that Vip3Aa binding with *Sf*-FGFR may initiate apoptosis pathways (Jiang et al., 2018a). The other studies also supported that Vip3Aa proteins triggered apoptosis (Hernández-Martínez et al., 2013; Osman et al., 2019).

III. *Spodoptera frugiperda*- Scavenger Receptor Class C like Protein (*Sf*-SR-C)

The affinity method combined with HPLC-MS/MS revealed the binding of biotin-labeled Vip3Aa protein with *Sf*-SR-C receptor on Sf9 cells *ex vivo* and *in vitro*. The down regulation of receptor expression in *S. frugiperda* and *S. exigua* reduced Vip3Aa toxicity. Simultaneously, heterologous expression of this scavenger receptor in the midgut of *Drosophila melanogaster* appreciably increased Vip3Aa virulence against larvae of *D. melanogaster*. This receptor showed only 27% identity with the scavenger receptor of *D. melanogaster.* There are four domains present in the extracellular *Sf*-SR-C: MAM (meprin, A-5 protein, and receptor protein-tyrosine phosphatase mu) domain; CCP (Complement control protein) domain; Ser/Thr rich domains, and Somatomedin B domain. It was speculated that only two domains (MAM and CCP) interact with protoxin Vip3Aa.

This protein exerts its toxicity effects *via Sf*-SR-C-mediated endocytosis (Jiang et al., 2018b). The super imposition of domain II of Vip3Aa11 protein (Jiang et al., 2020) and Vip3B2160 protein of Zheng et al. (2020) demonstrated the essential steps involved in the function of Vip3 as (1) cleavage of domain I from domain II and (2) conformational changes in domain II. A “membrane insertion model” was proposed by this group, recently (Jiang et al., 2020). Although the ion channel formation model is most acceptable for the activity of Vip3Aa proteins, the endocytosis activity of *Sf*-SR-C and apoptosis activity of FGFR and S2 proteins may also be responsible for its toxicity. Since this model is based upon experiments with Sf9 cells derived from ovaries, experiments using target insect’s midgut cells may provide further insight in respect of endocytosis and apoptosis models. The trigger of the apoptotic process has been observed in response to treatment with Vip3Ca proteins after activation of some caspases (Hernández-Martínez et al., 2013). Hence, recently, models related to the insecticidal activity of Vip3A proteins have been illustrated by Chakrabarty et al. (2020) and Syed et al. (2020).

A new insecticidal action mechanism (Oligomer formation) for Vip3 proteins was also demonstrated in that after the proteolytic process, the 65 kDa and 19 kDa portions of Vip3Aa form the complex of >240 kDa. The substitution of Serine at position 164 with Alanine or Proline inhibited the formation of this complex and loss of toxicity against *S. litura.* But substitution with Threonine did not affect the formation process and reduced the insecticidal activity by 35% which indicates that S164 is a critical amino acid position for the formation of >240 kDa protein complex and insecticidal activity (Shao et al., 2020).

### Spring-Loaded Mechanism of Vip3 Activation

A high-resolution architecture and activation mechanism of Vip3 has been proposed through Cryo-electron microscopy at 2.9 Å (Núñez-Ramírez et al., 2020). The spring-like apex of the N-terminal (four-helix coiled) converts into a long needle by a similar mechanism as proposed for influenza haemagglutinin, which also able to penetrate lipid bilayer (Carr and Kim, 1993; Blijleven et al., 2016). It was proposed by Núñez-Ramírez et al. (2020) that after insect ingestion and protease digestion in the midgut, the secreted tetrameric Vip3 protoxin interacts with the receptors present on the epithelial membrane. A pocket is formed between the tetramer body and domain III and the N-terminal of protein separates through this pocket due to interaction between the C-terminal of protein and receptors. Further, the protease digestion destabilizes the apex, which leads to the formation of an N-terminal long coiled-coil that penetrates the lipid bilayer. However, there is a scope for further understanding of the mechanism responsible for cell permeation and recognition of membrane receptors.

**FIGURE 5:**
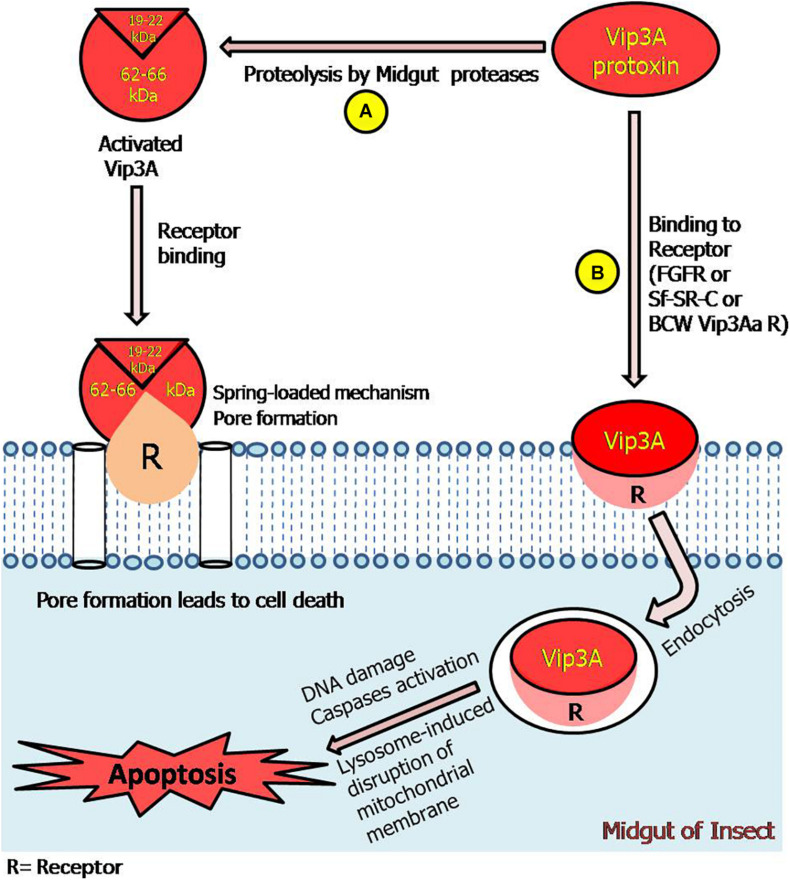
Model representing Vip3A proteins-insecticidal mechanism through pore formation and apoptosis. **(A)** The ∼62–66 kDa fragments along with ∼19–22 kDa fragment can bind with receptors leading to pore formation and cell death. **(B)** This represents that Vip3A-protoxin can bind to all three specific receptors in the target insect. Thereafter, enter the cell through receptor-mediated endocytosis and induced activation of caspases, DNA damage, and lysosome-induced disruption of the mitochondrial membrane which leads to apoptosis process in Sf9 cells and ultimately insect mortality. However, the responsible signaling pathway is still undefined.

In view of all these studies, a representative model of action mechanism for Vip3 protein has been proposed in this review (Figure 5).

#### Initial Response of Insects Upon Exposure to Vip3 Proteins

In recent years, researchers have been concerned about the overall understanding of the response of insects upon challenge by Vip3 proteins. Therefore, the immune responses and molecular interactions with target insect cells after treatments with *Bt* toxic proteins have been investigated to understand transcriptomic changes and other mechanisms such as autophagy, apoptosis, and endocytosis.

##### Transcriptomic alterations

To investigate larval response at the transcriptional level, Bel et al. (2013) performed genome-wide transcriptional analysis and studied differential gene expression in the larvae of *S. exigua* (8 and 24 h) after feeding with sublethal concentrations of Vip3Aa. The evaluation revealed that 5526 unigenes (19% of *S. exigua* unigenes) were regulated transcriptionally after Vip3Aa treatment, which was much higher in comparison to transcriptional variations in response to Cry proteins treatments in Dipteran (∼7%; Buchon et al., 2009); Coleopteran (∼1%; Oppert et al., 2012) and Lepidopteran (1–11%) pests (Eum et al., 2007; Huang et al., 2009; Wu et al., 2011). The numbers of genes expressed were similar, as numbers of down-regulated genes were 1,878 and 1,800 and up-regulated genes were 2,243 and 2,323 at 8 h and 24 h post toxin treatment, respectively, which was also contrasting in terms of treatment with of Cry proteins where up-regulated genes were lesser than the down-regulated genes (Van Munster et al., 2007; Oppert et al., 2012). In the study of Bel et al. (2013), the repression level of the down-regulated gene was observed as ∼ 660-fold which was higher than the ∼ 160-fold overexpression of up-regulated genes. In general, the overexpression of genes related to the immune response after intoxication with Vip3Aa including genes for recognition of pathogen; antimicrobial effectors (antimicrobial peptides (AMPs) and lysozymes); melanization was observed by this group, however, genes responsible for signaling pathways were not found to be regulated. AMP diapausin expression level was maximum (17–45-fold) followed by lebocin and gloverin. Along with these, B and D subfamilies of cercosporin AMPs were also regulated after Vip3Aa ingestion by *S. exigua*. The transcriptomic induction of AMPs and lysozymes in the larval midgut with the intoxication of sub-lethal doses of *Bt* toxins including Vip3Aa has been conducted (Crava et al., 2015).

Although the function of another class of immune-responsible Hdd family protein is still not defined, HDD1 and Hdd3 proteins from this family were also overexpressed after feeding. In addition to these, the 29 *repat* genes (REPAT: response to pathogen proteins) were also up-regulated after Vip3Aa ingestion by *S. exigua*, which validated the contribution of these genes in midgut response to Vip proteins. These REPATs proteins (midgut-infection responsive glycoproteins were discovered in *S. exigua* after *Bt* toxin and baculovirus intoxication and they seem to be involved in transcriptional activation of genes as a response to changes in midgut due to Vip/Cry proteins ingestion (Herrero et al., 2002; Herrero et al., 2007).

Among down-regulated transcripts, Apolipophorin was slightly down-regulated after Vip3Aa ingestion (Bel et al., 2013), which was in contrast with Cry proteins (Oppert et al., 2012; Contreras et al., 2013). Their down-regulation may be explained as they are lipid transporters and due to feeding inhibition after toxin ingestion, there could be an overall reduction in metabolic processing which results in a low level of apolipophorin. Along these, arylphorin (responsible for damaged cell replacement and proliferation) proteins were also found to be down-regulated in their study contrasting to Cry protein intoxication (Guo et al., 2012).

There were no changes in expression of ribosomal protein S2, and X-tox-like proteins mainly involved in the Vip3A mode of action besides this that two and nine genes among unigenes were shown homology with these proteins, respectively. There were no significant changes in the expression of genes responsible for the mode of action by *Bt*, which suggests that these are not involved in the action mechanism of Vip proteins. The difference in transcriptional response between Vip and Cry proteins feeding could be explained through their diverse action mechanisms (Bel et al., 2013). In conclusion, the up-regulated genes were responsible for various biological processes like hormonal regulations (JH binding proteins); immune response (repat family) and detoxification (glutathione S-transferase), and down-regulated genes were responsible for the encoding of enzymes serine protease (digestive); chitin deacetylase (increase in the peritrophic membrane permeability) and cytochrome P450 (oxidoreductase reactions). Another putative REVIP (REsponse-to-Vip intoxication) protein was discovered by Bel et al. (2013), which did not show homology with other proteins in any insect order.

Another study (Song et al., 2016) reported the up-regulation of PRPs (pattern recognition proteins) such as hemolin, β GRPs (β-1,3-glucan recognition proteins), CTLs (C-type lectins), and SCRs (Scavenger receptors) and down-regulation of PGPRs (peptidoglycan recognition proteins) in larvae of *S. litura* challenged with Vip3Aa intoxication. The genes related to signal transduction pathways, *i.e*., Toll, MAPK (Mitogen Activated Protein Kinase), and JAK/STAT (janus kinase/signal transduction and activator of transcription) have been also identified during transcriptional profiling. In agreement with Bel et al. (2013); after transcriptional profiling of 47 genes; another group also confirmed the up-regulation of immune responsive genes and down-regulation of digestive enzyme genes in larvae treated with three different doses of Vip3Ca proteins (Hernández-Martínez et al., 2013).

##### Endocytosis

However, in *Caenorhabditis elegans*, the endocytosis process is a kind of defensive approach by worms against the treatment of Cry5 toxins (Los et al., 2011). In the case of Vip proteins, this has been validated that contrary to earlier reports of inactivated Vip3 protoxins; the complete Vip3Aa protein could bind with *Sf*-SR-C (Scavenger Receptor-C) and receptor-mediated internalization of toxin *via* endocytosis (dynamin-dependent and micropinocytosis pathways) was responsible for the exertion of its toxicity effect on Sf9 cells derived from ovaries of *S. frugiperda* (Jiang et al., 2018b; Figure 5). The authors demonstrated the importance of endocytosis using endocytosis inhibitor dynasore, as there was complete inhibition and a clear effect on the toxicity of Vip3Aa proteins. It was also confirmed that compared to the CCP domain; the MAM domains more important during the endocytosis process. It will be interesting to observe the effect of Vip3 toxin on endocytosis in target insect’s midgut cells.

Recently, another group also studied the intracellular localization through laser scanning confocal microscopy using fluorescently labeled Alexa488-actVip3Aa. They confirmed that instead of the clathrin-or endocytic–dependent pathway by using early endosome marker Rab5, the activated Vip3Aa protein internalized through receptor-mediated endocytosis, which further interacts with ribosomal protein S2. This microscopic visualization also showed that activated toxin does not directly target the mitochondria in the cytosol but after internalization, resulted in disruption of cell division/differentiation and initiates apoptosis mediated cell death (Nimsanor et al., 2020). The interaction between protoxin Vip3Aa and ribosomal S2 protein was observed earlier also (Singh et al., 2010). Thus, these reports suggest endocytosis may be involved in the toxicity of Vip3 proteins.

##### Apoptosis

Three studies have proposed that target insect cells may respond to Vip3 proteins through the apoptotic process. In this regard, the first study reported that pro-Vip3Aa protein treatment causes cell arrest at the G2/M phase of Sf9 cells from immature ovaries of *S. frugiperda* pupae and induced apoptosis, which was demonstrated to be mediated through mitochondrial and *Sf*-caspase-1 dependent pathway. There was disruption of mitochondrial membrane potential in Vip3 protein treated Sf9 cells resulting in cell death (Jiang et al., 2016).

Another experiment conducted by Hernández-Martínez et al. (2013) demonstrated the cell death in *S. exigua* larvae due to triggered insect response; APN shedding and apoptosis after exposure to sub-lethal doses of Vip3 proteins. The apoptotic cell death was studied by analyzing the expression profile of 5 caspases genes. The *in vivo* trigger of apoptosis of midgut epithelial cells of *S. exigua* after treatment with Vip3Aa and Vip3Ca proteins was shown. Portugal et al. (2017) suggested that apoptosis may induce in the case of crystal proteins. Therefore, there may be a possibility of a similar mechanism in the Vip3 proteins, however, there is a need to expand our understanding of induced signaling pathways that lead to apoptosis.

The role of lysosomes in apoptosis was first mentioned by Duve and Wattiaux (1966). Later, the theory of the lysosome-mitochondria axis was taken into consideration as it highlighted that lysosomes release the hydrolyzing enzymes after an increase in the permeability of lysosomal membranes, which, subsequently leads to the release of cytochrome C; activation of caspases, and mitochondrial dysfunction. In addition to this, the role of Cathepsin D has been also demonstrated, as it is responsible for activation of Bax, which after translocation to mitochondria, leads to pore opening in the mitochondrial membrane, resulting in the release of apoptosis factor *viz*. cytochrome C (Guang et al., 2012).

Recently, efforts have been made to dissect the mitochondrial-mediated pathway of apoptotic cell death, which has revealed the importance of lysosomes in this process (Hou et al., 2020). The toxin induces the build-up of reactive oxygen species, cytochrome release, disruption of mitochondrial membrane potential, and activation of caspases (3 and 9) resulting in morphological changes (swelling and distorted cristae); overall dysfunctioning of mitochondria and finally reduced the viability of Sf9 cells. It was also established that the apoptosis process may also occur in intestinal epithelial cells of insects and the Bcl-2 protein family and caspases are the major regulators of programmed cell death. The activity of caspases-3 and caspases-9 was consistent in Sf9 cells treated with Vip toxins, which supported the induction of apoptosis through an intrinsic mitochondrial pathway. Along with mitochondrial damage, this study elucidated the effect of the toxin on morphological and physiological properties of lysosomes resulting in deformed lysosomes. These authors have proposed the role of cathepsins-L and D during apoptosis. It has been further proposed that lysosomes induce mitochondrial disruption and apoptosis while mitochondrial pathways facilitate the Vip3 protein-induced apoptosis process.

##### Autophagy

The insects also respond through autophagy for the maintenance of cell functions by removing damaged cell organelles and improperly folded proteins and there is a cross-network between apoptosis and autophagy. Autophagy involves (a) formation of autophagosome; (b) degradation of autophagosomes (Dodson et al., 2013; Mukhopadhyay et al., 2014). Lysosomes are the site of autophagosome degradation (Saftig and Klumperman, 2009).

It has been reported that Vip3 protein attacked the lysosome, reduced its membrane stability, and hampered the degradation of the autophagosome (Hou et al., 2020). The Vip3Aa-induced autophagy in Sf9 cells of *S. frugiperda* was also confirmed through the presence of autophagic vacuoles and increased level of protein Atg8-II (Hou et al., 2021). It was found that autophagy in Sf9 cells caused by this toxic protein was mediated through the AMPK-mTOR-ULK1 pathway associated with disruption of ATP-homeostasis in treated cells. It was also noticed that after 12 h of treatment, there was lysosomal degradation leading to the accumulation of autophagosome as this toxin impaired the autophagy flux. The damaged mitochondria and lysosomes were seen in autophagosomes, which speculated the role of autophagy in the degradation of damaged organs. However, with prolonged treatment, there was an accretion of the damaged lysosomes. This study suggested that with increased concentration of Vip3Aa, the concentration of degraded lysosomes is also increased, which causes the build-up of autophagosomes. It was proposed that the Vip3 protein-induced autophagy may be a kind of strategy to develop resistance in insects because autophagy can antagonize the virulent effect of Vip toxins. Thus, autophagy may play a role in the development of resistance in target insects due to delay in cell death and antagonistic effects.

### Vip4 Proteins

The *vip4Aa1* gene is 2895 bp long with 965 residues of deduced amino acid sequence. *In silico* analysis proposes its molecular mass of 108 kDa (Palma et al., 2014; Chakroun et al., 2016). Its predicted protein sequence shows 34% amino acid identity to Vip1Aa1 protein (Palma et al., 2014). Analysis of protein sequence revealed a signal peptide sequence (1–28) as well as two conserved domains: PA14 domain: 45–179 aa (an anthrax protective antigen) and a Binary_ToxB exotoxin bacterial domain (218–631 aa) usually present in binary Vip1. The insecticidal activity and host range of Vip4Aa1 remain unidentified, although this protein is found to be phylogenetically much more closely associated with Vip1 proteins than Vip2 and Vip3 proteins (Palma et al., 2014). In the modern nomenclature system, this protein is renamed as Vpb4 and put together with Vbp1 proteins in pesticidal proteins group Vpb (Crickmore et al., 2020). Recently, another 937 aa long Vbp4-type protein Vbp4Da2 (Accession No. AZJ95709) has been identified from *Bt* strain EG6657. The Vpb4Da2 protein has four domains: PA14 domain; Binary_toxB; toxB_2 and toxB_3 domains. Earlier target pests for Vip4/Vbp4 proteins were not known but, Vpb4Da2 protein is reported to be toxic against *Diabrotica virgifera* (Western corn root worm). The protein drastically reduced the beetle emergence (>97%) and provides root protection to maize transgenic plants in field conditions. This protein was also effective in controlling WCR populations resistant to Cry34Ab1/Cry35Ab1; DvSnf7 RNA and Cry3Bb1 resistant (Yin et al., 2020).

#### Identification of *vip3*-Type Genes in *Bt*

Initially, bioassays and biochemical testing approaches were used for isolation and characterization of various *Bt* strains, but these have been substituted by a widely used rapid and reliable molecular technique, *i.e*., polymerase chain reaction (PCR) testing for particular toxin signatures. This PCR technique amplifies the precise DNA fragments to find out the presence/absence of a target gene. *Bt* toxic genes identified by PCR can predict the insecticidal property of a given strain (Kuo and Chak, 1996; Porcar and Juárez-Pérez, 2003). Previous studies also screened *Bt* isolates from diverse ecological regions of different countries. In 1996, Estruch *et al.*, were able to detect only 60 (13%) isolates positive with *vip*3A-type genes, out of 463 isolates using 1.2 kb *vip3* fragment as a probe. Rice (1999) observed 652 bp *vip3A* gene in 29 (23.2%) (comprising about 20 *Bt* serovars) out of 125 *Bt* strains. In 2002, Loguercio *et al.*, screened only 12 *Bt* isolates and all were found positive for amplicon sizes of 150 bp and 1210 bp with two sets of *vip3*-specific primers, and further confirmed their identity as *vip3A* after sequencing. One other study reported 6 (37.5%) out of 16 *Bt* isolates found positive for *vip3A* type gene of 536 bp size (Guttmann and Ellar, 2000). Espinasse et al. (2003) detected 678 bp-sized *vip3*-type partial genes in 66 (52.8%) of 125 isolates studied.

In 2004, Arrieta and coworkers screened 105 *Bt* isolates from the coffee plantation, Costa Rica, and found that 78 (74.28%) contained *vip3* like gene (Arrieta et al., 2004). Mesrati et al. (2005) studied 256 *Bt* isolates and only 30% were found positive with a *vip3*-type amplicon of 419 bp. They also found HD1 reference strain positive for this gene. Another study screened the 24 *Bt* reference strains from BGSC (Bacillus Genetic Stock Center, Columbus), United States for *vip* homologs with primers having amplicon size of 2.4 kb and they reported 8 (33.33%) out of 24 *Bt* serovars were positive. The *vip3*-gene screening was performed by PCR amplification, using two primer sets having amplicon sizes of 364 bp and 444 bp, to screen more than 300 isolates and the majority of these isolates were found positive for *vip3*-like genes (Fang et al., 2007). A total of 382 (63.03%) out of 606 isolates were found to be positive with 1456 bp long *vip3A* gene (Liu et al., 2007).

Another study confirmed the presence of *vip*-like genes of 1621 bp size in 164 (87%) isolates among 188 Australian *Bt* isolates which was highest among all reported studies (Beard et al., 2008). The researchers reported that 82.6% of Iranian collection of 70 *Bt* isolates from 3 agro-climatic zones possessed the 1000 bp long *vip3Aa*-gene (Seifinejad et al., 2008). Out of 507 Spain and Bolivian isolates, 284 (48.9%) were found to be positive for 1621 bp long *vip3* gene, PCR amplified with degenerate primers (Hernández-Rodríguez et al., 2009). Sellami et al. (2013) also screened 212 *Bt* isolates from different ecological regions of Tunisia and 65 (30%) isolates and reference strain HD1, HD133 were found positive with *vip3*gene-specific primers (amplicon size varied between 0.67 to 2.13 kb). A large set of 2,134 *Bt* isolates from different ecological regions of Sichuan basin, China were screened with three pairs of primers and on average 67.4% of strains were detected with the presence of *vip3*-type gene (Yu et al., 2011b).

Thirty *Bt* isolates from different locations of Kashmir, India were screened with primers of Selvapandiyan et al. (2001) and 43.18% were found positive for *vip3*-type genes (Lone et al., 2016). Likewise, 8 (5.33%) out of 150 Indian *Bt* isolates were positive for 675 bp long *vip3*-gene (Rangeshwaran et al., 2016). Out of 15 *Bt* isolates collected from diverse habitats in 14 different locations of Assam, India, 40% of isolates were found positive for 1.4 kb size *vip3*-gene (Rabha et al., 2018). The other studies reported three (37.5%) out of eight isolates recovered from the arid environment of western Saudi Arabia to be positive for the presence of 2.3 kb *vip3A* gene (Abulreesh et al., 2012); 95 (69.3%) from 137 Algerian isolates of three geographical locations (Semi-arid, Desert and Mediterranean) possessed 1395 bp *vip3* gene (Djenane et al., 2017); 42% of 21 Sri Lankan isolates with 1029 bp *vip3A* gene (Baragamaarachchi et al., 2019) and 47.55% (243) of 511 Thailand isolates from various geographical regions were confirmed to be positive with 1591 bp long *vip3A* gene (Boonmee et al., 2019). The *vip3*-distribution in *Bt* isolates recovered from diverse habitats of different countries has been compiled in Table 2.

**TABLE 2:** *vip3*-type gene distribution in *Bt* isolates from different countries.

S. No.	Country	Isolates (number)	Gene size (bp)	% Gene frequency	Isolation source	References
1	United States	125	652	23.32	Soil and grain dust	Rice (1999)
2	India	49	700	2	Soil	Selvapandiyan et al. (2001)
3	Brazil	12	150, 1210	100	Soil	Loguercio et al. (2002)
4	France and 31 countries of 5 continents	125	678	52.8	Soil, plants, animal waste, dust, insects, etc.	Espinasse et al. (2003)
5	Costa Rica	105	−	74	Soil, Foliage, Leaf litter	Arrieta et al. (2004)
6	Costa Rica	146		54	Soil, leaf litter, fresh leaves, other material of forest area	Arrieta and Espinoza (2006)
7	Tunisia	256	419	30	Soil	Mesrati et al. (2005)
8	China	606	1456	63.03	Not mentioned	Liu et al. (2007)
9	Australia	187	1621	87	Soil, bird nest, and grain dust	Beard et al. (2008)
10	Iran	70	1000	82.6	Soil, leaf samples, and dead insects	Seifinejad et al. (2008)
11	Spain and Bolivia	507	1621	48.9	Soil from Agricultural, Mountain, Wetland and Forest, fodder and grain from silos and mills, olive factory, Tuber warehouse, Insects, damaged crops, wine factory, dung, etc.	Hernández-Rodríguez et al. (2009)Hernández-Rodríguez and Ferré (2009)
12	Tunisia	212	670–2370	30	Soil	Sellami et al. (2013)
13	China	2,134	364, 444	67.4	Soil from Mountain, Forest Farmland and snowcapped mountain	Yu et al. (2011b)
14	Western Saudi Arabia	8	2300	37.5	Agricultural and urban soil, dead larvae	Abulreesh et al. (2012)
15	India	39	1113	12.5%	Not mentioned	Shingote et al. (2013)
16	India	44	700	43.18	Lake sediments, forest soil, and maize field	Lone et al. (2016)
17	India	150	675	5.33	Soil/infected insects	Rangeshwaran et al. (2016)
18	Argentia	268	608	91.3	Soil, spider web, leaves, dust, dead larvae	Sauka and Benintende (2017)
19	Algeria	137	1395	69.3	Lake sediments, rhizospheric and non-rhizospheric soil, dead insects, and stored grain	Djenane et al. (2017)
20	India	15	1400	40	Soil from tea and rice field	Rabha et al. (2018)
21	Turkey	80	1395	23	Soil, fruits, and fig leaves	Şahin et al. (2018)
22	Sri Lanka	21	1029	42	Soil	Baragamaarachchi et al. (2019)
23	Thailand	511	1591	47.55	Soil	Boonmee et al. (2019)

#### Cloning, Characterization, and Toxicity Potential of *vip3*-Genes

In the year 1996, novel *vip3A(a)* gene and *vip3A(b)* gene were isolated from *Bt* strains AB88 and AB424, respectively, and their proteins showed different levels of larvicidal activity against *A. ipsilon*, *S. exigua*, *S. frugiperda*, *Heliothis virescens* and *Helicoverpa zea.* The accession numbers L48811 [*vip3A(a)*] and L48812 [*vip3A(b)*] were assigned by NCBI GenBank after their sequence submission. The deduced amino acid sequence of these genes predicted a protein of 88.5 kDa (791 aa), which did not show homology with existing proteins and was reported as a novel class of lepidopteran-specific toxins (Estruch et al., 1996). Donovan et al. (2001) observed that *vip3Aa1* gene deletion from a *Bt* strain reduces the insecticidal activity and proposed that toxicity of these strains have been caused by Vip3. The three genes *vip14*, *vip15*, and *vip83* were identified in isolates Y*BT*-1416, Y*BT*-1535, and Y*BT*-833, respectively. The gene *vip83* (Vip3Aa7) was expressed into the pHT315 vector and its protein was reported toxic against larvae of lepidopteran insect (Cai et al., 2002).

Doss et al. (2002) constructed the *Bt* subsp. *kurstaki* (*Bt*. *k*.) genomic library and characterized a positive clone having the full-length gene, *vip3V* (Vip3Aa10) and pET-22b (+) expression vector was used for its sub-cloning and over expression in *E. coli*. An anion-exchange chromatographic method was developed for elution of purified protein, which demonstrated toxicity against several lepidopteran larvae, but it was not toxic against the mosquito (*Culex quinquefasciatus)* and silkworm (*Bombyx mori*) larvae. A full length 2.37 kb *vip3A-S184* (*vip3Aa13*) gene was cloned and expressed into pGEMT-Easy and pQE30 vector. The western blot confirmed the presence of 89 kDa protein. It was examined through Transmission Electron Microscopy that only 19% of this protein was soluble and the rest fraction was insoluble as inclusion bodies (Chen et al., 2002). The *vip3Aa14* gene isolated from *B. thuringiensis* subsp. *tolworthi* was expressed in pET29a expression vector, induced for 16–18 h at 15°C using 1 mM IPTG, and both truncated (without signal peptide) and full-length proteins were reported to be highly toxic against *Plutella xylostella* and *S. litura* (Bhalla et al., 2005).

Another novel gene *vip3LB* (Vip3Aa16) from *Bt* strain BUMP95 has been isolated and reported to be toxic against *Ephestia kuehniella*. For the first time, it was shown that the large plasmid (31.8 kb) carries the *vip*3LB gene which also contained the *cry1Ia* genes of *Bt*. The sequence of 789 amino acids sequence was predicted and SDS PAGE-analysis confirmed the presence of 88.5 kDa protein. It possessed dissimilarity with the other *vip3*-type genes (Mesrati et al., 2005). Fang et al. (2007) screened their collection of *Bt* isolates for identification of novel *vip3* genes by PCR-based approach. A novel *vip3* gene was cloned and developed a chimeric gene by sequence swapping with *vip3Aa1* gene. They found that one chimeric Vip3 toxin gained novel properties of insecticidal activity, which was highly active against a *Bt*-resistant strain of *Trichoplusia ni*. Liu *et al.*, reported two novel genes encoding Vip3Aa11 (Liu et al., 2004) and Vip3Aa19, out of which Vip3Aa19 showed insecticidal activity against *H armigera*, *S. exigua*, and *P. xylostella* larvae. Vip3Aa11 was found toxic against *H. armigera* and *S. exigua*. They also reported a novel gene, which showed 83% sequence identity with *vip3Af1* gene (Liu et al., 2007). The novel gene *vip3Bb2* (94% identical with *vip3Ba1*) was cloned from *Bt* isolate C81 from the Australian *Bt* collection. The culture supernatant was reported to be toxic against *H. armigera* (Beard et al., 2008). In 2011, the Vip3Aa16 was also reported toxic against *S. littoralis* and it was confirmed that *E. kuehniella* was more susceptible (8.5 times) than *S. littolaris* (Mesrati et al., 2011). Further experiments suggested that the Vip3Aa16 protein can remain stable in culture supernatant at the later sporulation phase and it exhibited toxicity against 2nd instar of *S. littolaris* (Sellami et al., 2011).

Another research group reported a method for characterization of *vip3A* genes through direct sequencing of PCR product amplified using gene-specific primers and evolutionary analysis to discover novel Vip protein genes of *Bt* isolates, to improve the prospects for insect control (Asokan et al., 2012). The novel *vip3*-kind genes were isolated from the Spanish *Bt* collection and sequence analysis revealed a new class of Vip3C proteins and these proteins were named Vip3Ca1, Vip3Ca2, and Vip3Ca3. The bioassay confirmed that Vip3Ca3 exhibited >70% mortality after 10 days against larvae of four lepidopteran pests (Palma et al., 2012). The coding region of the *vip3Aa* gene (2.37 kb) was isolated, cloned, and expressed in expression vector pQE-30, which revealed 98% sequence homology with available *vip3Aa* genes. The expressed protein was not observed in the pellet but was reported to be present only in concentrated supernatant, which confirmed its secretion into the culture medium. SDS-PAGE analysis and western blotting of supernatant confirmed the presence of 89 kDa band of Vip3Aa protein. They found this protein toxic against *S. littolaris* with LC_50_ value 142.4 μg/ml (El-Ghareeb et al., 2012). It was demonstrated that deduced amino acid sequence of Vip3Ad, Vip3Ae, and Vip3Af shared 85%, 81%, and 88% identity with Vip3Aa, respectively. It was reported that Vip3Ae and Vip3Af were highly toxic as compared with Vip3Aa in both forms, as protoxin or activated toxin, against *S. frugiperda*. Vip3Ae protoxin was highly toxic against *A. ipsilon* than protoxin forms of other proteins. However, Vip3Ad did not have larvicidal activity against both species (Hernández-Martínez et al., 2013).

Two novel genes, Vip3Aa45 (789 aa) and Vip3Ag4 (787 aa) were identified from the Spanish *Bt* collection. The pairwise identity of 82% was observed between them. The proteins expressed from both genes showed higher toxicity for *S. littoralis* and *Lobesia botrana* followed by *S. exigua*. Vip3Aa45 was found more toxic against *Mamestra brassicae* and Vip3Ag4 against *Chrysodeixis chalcites* (Palma et al., 2013). The protein expressed from partial *vip3*-type genes (1113 bp) identified in isolates PDKV-08, PDKV-21, NCIM-5110, and NCIM-5132 exhibited lower toxicity than the reference strain HD-1 against *H. armigera* (Shingote et al., 2013). The *vip3-*gene from *Bt* supsp. *aegypti* isolate C18 was identified and the insecticidal activity of both pellet and supernatant was demonstrated against *A. ipsilon.* Their bioassay study revealed that pellet from 48 h culture growth was more toxic against this pest. The pellet lost its activity after autoclaving and boiling but remained stable at very high temperatures (Osman et al., 2013). The bioassay study to analyze the toxic potential of Vip3Aa and Vip3Ae revealed that protoxin Vip3Aa was 12 times more toxic against *S. frugiperda* than *S. exigua*. These proteins were equally toxic to *S. frugiperda* but Vip3Ae was more toxic for *S. exigua* (Caccia et al., 2014). Vip proteins were also evaluated for their toxicity potential against whiteflies. Two genes isolated from *Bt* isolates C-18 and DI-29 were expressed and C-18 was reported to be more toxic with LC_50_ value of 90 ppm as compared with other isolates with LC_50_ of 160 ppm (El-Gaied et al., 2014).

In 2015, sub-lethal effects of Vip3A-proteins were accessed as it affected the developmental and reproductive behavior of *P. xylostella* and *H. virescens*. The larval and pupal development period was increased in treated insects. The emergence of pupa and adult was also lower compared to that in larvae fed on a control diet (Gulzar and Wright, 2015). Vip3A proteins were reported to be toxic against *S. exigua*, *S. frugiperda*, and *A. ipsilon*, which are reported to be less susceptible to crystalline proteins. However, Vip3Aa1 and Vip3Aa14 proteins were not toxic to *H. armigera* but the laboratory and field populations of *H. virescens* and *H. zea* showed susceptible nature of different degrees (Chakroun et al., 2016). The *vip3A* gene identified from *Bt*-EG1 isolate and its crude protein extract was reported to be more toxic to *P. xylostella* (LC_50_: 0.43 μg/ml) than *S. litura* (LC_50_: 5.83 μg/ml). However, the crude protein extract was not toxic to *H. armigera* (Rangeshwaran et al., 2016). The Vip3Aa protein isolated from *Bt* isolates WB5 exhibited higher insecticidal activity against *S. litura* compared to that against *H. armigera* and *S. exigua*. This study demonstrated that with increase in larval age, insecticidal activity of Vip3Aa protein decreases statistically and was explained with LC_50_ values: 2.609 ng/cm^2^ (neonates); 28.778 ng/cm^2^ (first instar); 70.460 (second instar) ng/cm^2^ for second instar larvae, and 200.627 ng/cm^2^ (third instar). The electron microscopic examination showed damage in the midgut epithelial cells of treated larvae (Song et al., 2016). A novel gene *vip*3 (459), isolated from *Bt*-BLB459 was cloned into expression vector pET-14b and its expressed protein exhibited toxicity against *Agrotis segetum*, *S. littoralis*, and *E. kuehniella* (Boukedi et al., 2017). The toxicity potential of Vip3Aa protein was evaluated against 12 field populations of *H. armigera* collected from cotton fields of different geographical locations in China. The LC_50_ value was found between 0.05 to 1.311 μg/cm^2^. This study did not find cross-resistance of protein Vip3Aa in *H. armigera* strains, which were highly resistant to Cry1Ac and Cry2Ab (Wei et al., 2017).

Recently, a new allele of *vip3Aa* gene was isolated from *Bt* isolate 6A, whose protein was named Vip3Aa65 and was tested in comparison to the Vip3Aa16 protein. Vip3Aa16 was more toxic to species of *Spodoptera* than Vip3Aa65, while both toxins were similarly toxic against *H. armigera* and Peach moth *Grapholita molesta* (Şahin et al., 2018). A 2.37 kb full-length gene was identified in Indian native *Bt* isolate JK37 and named Vip3Aa61. This gene was cloned into a pET-28a(+) expression vector and the expression of 89 kDa protein was confirmed through SDS-PAGE analysis and western blot. The bioassay demonstrated LC_50_ value 169.63 ng/cm^2^ for the second instar stage of *H. armigera* (Lone et al., 2018). Two isolates Bn*Bt* and MnD from *Bt* subsp *kurstaki* were found positive for the presence of 1.62 kb *vip3* gene. The 90 kDa Vip3 protein was expressed and showed 10th-day mortality of 86.66% (Bn*Bt*) and 83.33% (MnD) in comparison to 13.33% in control larvae against the second instar stage of *S. littoralis*. The LC_50_ values were obtained as 55.15 and 41.86 ng/μl for MnD and Bn*Bt*, respectively (Güney et al., 2019). The assessment of the potential of Vip3 protein against whiteflies was done through isolation of complete coding sequence (2.4 kb) of gene from an isolate of Egypt. Vip3 protein expression and its molecular weight were confirmed by western blot and SDS-PAGE techniques. This study demonstrated that the whole-cell culture of Vip3 protein-expressing cells showed more toxicity potential (4.7 times higher LC_50_) against whiteflies than Vip3 protein alone. Therefore, these proteins can be successfully used in the control and management of whiteflies (El-Gaied et al., 2020). Recently, Vip3Aa19 protein was found to be very effective against *A. ipsilon* attack in maize with LC_50_ value of 0.43 μg/g as compared to chimeric protein Vip3_Ch1 (5.53 μg/g). The LC_50_ values were higher for Cry1Ac (184.77 μg/g) and Cry1F (83.62 μg/g), which indicates the better toxicity potential of Vip3 proteins as compared with the latter for this insect (Yan et al., 2020). Various modified and chimeric Vip3A proteins, either alone and in combination with crystalline proteins, developed and tested for their insecticidal activity have been described in Table 3.

**TABLE 3:** Chimeric Vip3A proteins alone or in combination with Cry proteins and effects on insecticidal activity against target pests.

S. No.	Modified/Interactive protein combination	Proteins involved	Technique used	Effect on toxicity	References
1.	Vip3AcAa	Vip3Ac1 (N-terminal) Vip3Aa1 (C-terminal)	Domain Swapping	*Ostrinia nubilalis* (GIA); *S. frugiperda, H. zea*, and *Bombax mori* (IIA); Cry1Ac resistant strain of *Trichoplusia ni* (GIA)	Fang et al. (2007)
2.	Vip3AaAc	Vip3Aa1 (N-terminal) Vip3Ac1 (C-terminal)	Domain Swapping	*S. frugiperda* and *H. zea* (RIA); *Bombax mori* (LIA)	Fang et al. (2007)
3.	Cry1Ac-Vip3	Cry1Ac Vip3Aa14	Protein fusion	*H. armigera* and *P. xylostella* (EIA as Cry1Ac); *S. litura* (RIA compared to Vip3Aa14)	Saraswathy et al. (2008)
4.	Cry1C-Vip3	Cry1C (promoter) Vip3Aa7 (truncated) Cry1C (C-terminal)	Protein fusion and change in promoter of gene	*P. xylostella*, *H. armigera*, and *S. exigua* (RIA)	Song et al. (2008)
5	Cry9C-Vip3	Cry9Ca (N-terminal) Vip3Aa7	Protein fusion	*P. xylostella* (IIA)	Dong et al. (2012)
6	Cry1Ac-Vip3	Cry1Ac (N-terminal) Vip3Aa16	Protein fusion	*E. kuehniella* (IIA)	Sellami et al. (2018)
7	Vip3Ab1-740	Vip3Ab1 (C-terminal)	Modified 177 aa at C-terminal	*S. frugiperda* (EIA); *S. eridania* (GIA);	Bowling et al. (2019)
8	Vip3A-Vip3C (Vip3_ch1)	Vip3Ca2 (N-terminal) Vip3Aa45 (Central Domain) Vip3Aa45 (C-terminal)	Domain Swapping	*O. furnacalis, Anticarsia gemmatalis* (LIA); *S. frugiperda, S. littoralis, M. brassicae, H. armigera* (RIA) comparative to Vip3Aa	Gomis-Cebolla et al. (2020)
9	Vip3A-Vip3C (Vip3_ch2)	Vip3Aa45 (N-terminal) Vip3Ca2 (Central Domain) Vip3Ca2 (C-terminal)	Domain Swapping	*S. frugiperda* (IIA compared to Vip3Ca) *O. furnacalis, M. brassicae, A. gemmatalis* (RIA)	Gomis-Cebolla et al. (2020)
10	Vip3A-Vip3C (Vip3_ch4) Double chimeric	Vip3Ca2 (N-terminal) Vip3Aa45 (Central Domain) Vip3Ca2 (C-terminal)	Domain Swapping	*S. frugiperda, S. littoralis, S. exigua, A. gemmatalis*, *H. armigera, M. brassicae* (LIA)	Gomis-Cebolla et al. (2020)
11	Vip3A-Vip3C (Vip3_ch5)	Vip3Aa45 (N-terminal) Vip3Aa45 (Central Domain) Vip3Ca2 (C-terminal)	Domain Swapping	*S. frugiperda, S. littoralis, S. exigua, A. gemmatalis*, *H. armigera, M. brassicae*, *O. furnacalis* (NIA)	Gomis-Cebolla et al. (2020)
12	Vip3_ch1	Vip3Ca2 (N-terminal) Vip3Aa45 (Central Domain) Vip3Aa45 (C-terminal)	Domain Swapping	*A. ipsilon* (RIA) comparative to Vip3Aa19	Yan et al. (2020)
13	Vip3_ch4	Vip3Ca2 (N-terminal) Vip3Aa45 (Central Domain) Vip3Ca2 (C-terminal)	Domain Swapping	*A. ipsilon* (RIA) comparative to Vip3Aa19	Yan et al. (2020)

*GIA, gain of insecticidal activity; IIA, increase in insecticidal activity; RIA, reduction insecticidal activity; LIA, lost insecticidal activity; EIA, equal insecticidal activity; NIA, no insecticidal activity.*

## Importance of *vip* Genes in Gene Pyramiding and Events Developed

The dissimilar characteristics from Cry proteins like different production-stage (vegetative) and different sequence and receptors (membrane binding sites) of Vip3 proteins have evinced interest of Agri-biotech companies *viz.* Syngenta, Bayer Crop Sciences, Monsanto, DuPont, and Dow Agrosciences for combining *vip3A* genes with *cry* genes, which have already been transferred in cotton and maize to control devastating insect pests and to include them in insect pest management strategies for the delay in the development of resistance in target pest populations. Various studies demonstrated the interaction between different kinds of Vip and Cry proteins. Their results explained about synergistic and antagonistic behavior of different combinations, which will be helpful in the selection of genes for stacking in transgenic crops (Table 4.) These different characteristics make Vip3 a useful complement for Cry proteins and also decrease the probability for the development of cross-resistance. Various GM events containing *vip3A* genes have been developed and released in several countries by these companies (Tables 5, 6). the A cotton cultivar VipCot^TM^ developed by Syngenta through stacking with *vip3A* and *cry1Ab* for management of major lepidopteran cotton pests: army worms, beet army worms, tobacco bud worms, and bollworms (Kurtz et al., 2007; Adamczyk and Mahaffey, 2008). The efficacy of VipCot^TM^ cultivars was tested and showed significant larval mortality of 97–100% against *H. zea* and *H. virescens* as compared with conventional cotton (Kurtz et al., 2007; Palekar et al., 2011). Cross-resistance to Vip3A protein was not observed in the resistant population of *H. zea* (AR) (Anilkumar et al., 2008). One transgenic line of rice was also developed against Asiatic rice borer (*C. suppressalis*) through the expression of Cry1Ab and Vip3Ah proteins, which resulted in 100% mortality on all developmental stages of the rice plant (Chen et al., 2010). Sellami et al. (2011) transformed *Bt* strains with *vip3A* genes and those strains exhibited 10-times higher oral toxicity against *S. littoralis* and *S. exigua* under lab conditions. The *vip3Aa20* gene alone and also with *cry1Ab* gene was incorporated in transgenic corn Agrisure Viptera and Agrisure Viptera 3, respectively, against *Spodoptera frugiperda* (Christou et al., 2006; Bernardi et al., 2016; Horikoshi et al., 2016). The genetically modified maize Agrisure Viptera 3111 (*cry1Ab + cry3A+ vip3Aa20*) with triple genes provides resistance to various maize primary pests, root worms, borers, and secondary pests (cutworms, armyworm, earworm, *etc*.) which are not susceptible to Cry proteins (Watkins et al., 2012). The high level of resistance against horn caterpillar, stem borer, and leaf folder has been found in genetically modified mega rice-cultivar Swarna (Marker-free transplastomic Swarna) which was modified with *Syn vip3BR* by engineering it in chloroplast genome using Cre/lox recombination technique (Pradhan et al., 2016).

**TABLE 4:** Synergistic and antagonistic behavior of Vip and Crystalline proteins.

S. No.	Expressed proteins	Target pest	References
**Synergism**			
1.	Vip3Aa29/Cyt2Aa3	*Chilo suppressalis*, *S. exigua*	Yu et al. (2012)
2.	Vip3Aa/Cry1Ia10	*Spodoptera albula*, *S. frugiperda* and *S. cosmioides*	Bergamasco et al. (2013)
3.	Vip3Aa/Cry9a	*O. furnacalis*, *Chilo suppressalis*	Wang et al. (2018)
4.	Vip3Ca/Cry1Ab	*O. furnacalis*, *Mythimna separata*	Yang et al. (2018)
5.	Vip3AcAa+Cry1Ac	Cry1Ac resistant *H. armigera*	Chen et al. (2018)
6.	Vip3Aa/Cry1Ab; Vip3Aa/Cry2Ab; Vip3Ca/Cry1Ea; Cry1Ab/Cry2Ab/Vip3Aa	*S. frugiperda*	Figueiredo et al. (2019)
**Antagonism**			
1.	Vip3Aa/Cyt2Aa	*Culex quinquefasciatus*	Yu et al. (2012)
2.	Cry1Ia10 + Vip3Aa	*S. eridania*	Bergamasco et al. (2013)
3.	Vip3A/Cry1A; Cry1Ca/Vip3Aa; Cry1Ca/Vip3Ae; Cry1Ca/Vip3Af; Vip3Af/Cry1Aa	*S. frugiperda*	Lemes et al. (2014)

**TABLE 5:** List of approved events containing Vip3A proteins in commercial *Bt* cotton in different countries.

S. No.	Event	Developer	Name of Countries and approval year	Countries No.	Trade Name	Genes	Target traits
1.	COT102 (IR102)	Syngenta	Australia (2005), Canada (2011), China (2015), Colombia (2016), Costa Rica (2017), Japan (2012), Mexico (2010), New Zealand (2005), Philippines (2015), South Korea (2014), Taiwan (2015), United States (2005)	12	VIPCOT^TM^ Cotton	*vip3A(a)*	Lepidopteran insect resistance
2.	GHB614 × T304-40 × GHB119 × COT102	Bayer Crop Science	Australia (2016), Brazil (2017), Japan, Mexico (2015), Philippines (2020), South Korea (2015), Taiwan (2016)	7	Twin Link Plus (Glytol^TM^ × Twinlink^TM^ × VIPCOT^TM^ Cotton)	*cry1Ab* + *cry2Ae* + *vip3A(a)* + *mepsps*	Herbicide tolerance and lepidopteran insect resistance
3.	COT102 × COT67B	Syngenta	Costa Rica (2009)	1	VIPCOT^TM^ Cotton	*cry1Ab* + *vip3A(a)*	Insect resistance
4.	COT102 × COT67B × MON88913	Syngenta and Monsanto	Costa Rica (2009)	1	VIPCOT^TM^ Roundup Ready Flex^TM^ Cotton	*cry1Ab* + *vip3A(a)* + *cp4 epsps*	Herbicide tolerance and Insect resistance
5.	3006-210-23 × 281-24-236 × MON88913 × COT102	Dow AgroSciences LLC	Japan (2013), Mexico (2014), South Korea (2014)	3	Wide Strike 3 (Widestrike^TM^ × Roundup Ready Flex^TM^ × VIPCOT^TM^ Cotton)	*cry1Ac* + *vip3A(a)* + *cry1F* + *cp4 epsps* + *pat*	Herbicide tolerance and lepidopteran insect resistance
6.	COT102 × MON15985	Monsanto	Australia, Japan, Mexico (2014)	3	Bollgard^®^ III	*cry1Ac* + *cry2Ab2* + *vip3A(a)*	Lepidopteran insect resistance
7.	COT102 × MON15985 × MON88913	Monsanto	Australia (2014), Brazil (2016), Japan (2014), South Korea (2015), Taiwan (2016)	5	Bollgard^®^ III × Roundup Ready^TM^ Flex^TM^	*cry1Ac* + *cry2Ab2* + *vip3A(a)* + *cp4 epsps*	Herbicide tolerance and lepidopteran insect resistance
8.	COT102 × MON15985 × MON88913 × MON88701	Monsa nto	Australia (2016), Brazil (2018), Colombia (2012), Japan (2016), Mexico (2015), South Korea (2016), Taiwan (2017)	7	Not available	*cry1Ac* + *cry2Ab2* + *vip3A(a)* + *cp4 epsps* + *dmo* + *bar*	Herbicide tolerance and lepidopteran insect resistance
9.	281-24-236 × 3006-210-23 × COT102	Dow AgroSciences LLC	Brazil (2018)	1	Not available	*cry1Ac* + *vip3A(a)* + *cry1F*	Lepidopteran insect resistance
10.	281-24-236 × 3006-210-23 × COT102 × 81910	Dow AgroSciences LLC	Brazil (2019), Japan (2016)	2	Not available	*cry1Ac* + *vip3A(a)* + *cry1F* + *pat* + *aad-1*2	Herbicide tolerance and Insect resistance
11.	3006-210-23 × 281-24-236 × MON88913 × COT102 × 81910	Dow AgroSciences LLC	Japan (2015), Mexico (2016), South Korea (2017)	3	Not available	*cry1Ac* + *vip3A(a)* + *cry1F* + *cp4 epsps* + *pat* + *aad-1*2	Herbicide tolerance and Insect resistance
12.	GHB811 × T304-40 × GHB119 × COT102	BASF and Bayer Crop Science	Brazil (2016)	1	Not available	*cry1Ab* + *cry2Ae* + *vip3A(a)* + *hppdPF W336* + *pat*	Herbicide tolerance and lepidopteran insect resistance
13.	T304-40 × GHB119 × COT102	Bayer Crop Science	Brazil (2018)	1	Not available	*cry1Ab* + *cry2Ae* + *vip3A(a)* + *bar*	Herbicide tolerance and lepidopteran insect resistance

*Compiled from ISAAA GM Approval Database (http://www.isaaa.org/gmapprovaldatabase).*

**TABLE 6:** List of approved events containing Vip3A proteins in commercial *Bt* maize in different countries.

S. No.	Event	Developer	Name of Countries and approval year	No. of countries	Trade Name	Genes	Target traits
1.	Bt11 × MIR162	Syngenta	Argentina (2014), Brazil (2015), Colombia (2016), European Union (2016), Japan (2010), Mexico (2017), Philippines (2013), South Korea (2016), Taiwan (2015)	9	Agrisure^®^ Viptera^TM^ 2100	*cry1Ab* (truncated) + *vip3Aa20* + *pat*	Herbicide tolerance and lepidopteran insect resistance
2.	Bt11 × MIR162 × GA21	Syngenta	Argentina (2011), Brazil Canada (2010), Colombia (2012), European Union (2016), Japan, Mexico (2010), Paraguay (2015), Philippines (2010), South Africa (2011), South Korea (2012), Taiwan (2011), Uruguay (2012)	13	Agrisure^®^ Viptera^TM^ 3110	*cry1Ab* + *vip3Aa20* + *mepsps* + *pat*	Herbicide tolerance and lepidopteran insect resistance
3.	Bt11 × MIR162 × MIR604 × GA21	Syngenta	Argentina (2012), Brazil (2014), Canada (2010), Colombia (2012), European Union (2016), Japan, Mexico, Philippines, South Korea (2010), South Africa, Taiwan (2011)	11	Agrisure^®^ Viptera^TM^ 3111, Agrisure^®^ Viptera^TM^ 4	*cry1Ab* + *vip3Aa20* + *mcry3A* + *mepsps* + *pat*	Herbicide tolerance and insect resistance (lepidopteran and coleopteran)
4.	Bt11 × MIR162 × TC1507 × GA21	Syngenta	Argentina (2014), Canada (2010), Colombia (2016), Japan (2010), Mexico (2011), Philippines (2010) South Korea (2012), South Africa, Taiwan (2011)	9	Agrisure^®^ Viptera^TM^ 3220	*cry1Ab* + *cry1Fa2* + *vip3Aa20* + *mepsps* + *pat*	Herbicide tolerance and lepidopteran insect resistance
5.	MIR162	Syngenta	Argentina (2011), Australia, Brazil (2009), Canada (2010), China (2014), Colombia, European Union (2012), Indonesia (2011), Iran (2016), Japan (2010), Malaysia (2016), Mexico (2010), New Zealand (2009), Paraguay (2014), Philippines (2010), Russia (2011), Singapore (2017), South Africa (2014), South Korea (2010), Taiwan (2009), Turkey (2015), United States (2008), Vietnam (2014), Zambia (2017)	25	Agrisure^TM^ Viptera	*vip3Aa20*	Lepidopteran insect resistance
6.	BT11 × MIR162 × MIR604	Syngenta	Brazil (2019), European Union (2016)	2	Agrisure^®^ Viptera^TM^ 3100	*cry1Ab* + *mcry3A* + *vip3Aa20* + *pat*	Herbicide tolerance and insect resistance (lepidopteran and coleopteran)
7.	5307 × MIR604 × Bt11 × TC1507 × GA21 × MIR162	Syngenta	Argentina (2018), Brazil (2015), Canada (2013), Colombia (2016), Japan Mexico (2013), South Africa (2014), Taiwan (2013)	8	Agrisure^®^ Duracade^TM^ 5222	*cry1Ab* + *cry1Fa2* + *ecry3.Ab* + *mcry3A* + *vip3Aa20* + *mepsps* + *pat*	Herbicide tolerance and insect resistance (lepidopteran and coleopteran)
8.	MON89034 × TC1507 × NK603 × MIR162 × DAS40278	Dow Agro Sciences LLC	Brazil (2018), Japan (2016), Mexico, South Korea, Taiwan (2018)	5	Power Core^TM^ × MIR162 × Enlist^TM^	*cry1Fa2* + *cry2Ab2* + *vip3Aa20* + *cp4 epsps* + *pat* + *aad-1*	Herbicide tolerance and lepidopteran insect resistance
9.	MIR162 × GA21	Syngenta	Argentina, European Union (2016), Paraguay (2015)	3	Not available	*vip3Aa20* + *mepsps*	Herbicide tolerance and lepidopteran insect resistance
10.	MIR162 × MIR604	Syngenta	Brazil (2019), European Union (2016)	2	Not available	*vip3Aa20* + *mcry3A*	lepidopteran and coleopteran insect resistance
11.	MIR162 × MIR604 × GA21	Syngenta	European Union (2016)	1	Not available	*vip3Aa20* + *mcry3A* + *mepsps*	Herbicide tolerance and insect resistance (lepidopteran and coleopteran)
12.	MIR162 × MIR604 × TC1507	Syngenta	Brazil (2019)	1	Not available	*vip3Aa20* + *cry1Fa2* + *mcry3A* + *pat*	Herbicide tolerance and insect resistance (lepidopteran and coleopteran)
13.	MIR162 × MIR604 × TC1507 × 5307	Syngenta	Brazil (2019)	1	Not available	*vip3Aa20* + *cry1Fa2* + *mcry3A* + *ecry3.1Ab* + *pat*	Herbicide tolerance and insect resistance (lepidopteran and coleopteran)
14.	MIR162 × MIR604 × TC1507 × 5307 × GA21	Syngenta	Brazil (2019)	1	Not available	*vip3Aa20* + *cry1Fa2* + *mcry3A* + *ecry3.1Ab* + *mepsps* + *pat*	Herbicide tolerance and insect resistance (lepidopteran and coleopteran)
15.	Bt11 × MIR162 × MIR604 × MON89034 × 5307 × GA21	Syngenta	Canada (2017), Japan (2016), South Korea, Taiwan (2018)	4	Not available	*cry1A.105* + *cry1Ab* + *cry2Ab2* + *mcry3A* + *ecry3.Ab* + *vip3Aa20* + *mepsps* + *pat*	Herbicide tolerance and insect resistance (lepidopteran and coleopteran)
16.	BT11 × MIR162 × MIR604 × TC1507	Syngenta	Brazil (2019)	1	Not available	*cry1Ab* + *cry1Fa2* + *mcry3A* + *vip3Aa20* + *pat*	Herbicide tolerance and insect resistance (lepidopteran and coleopteran)
17.	BT11 × MIR162 × MIR604 × TC1507 × 5307	Syngenta	Brazil (2019)	1	Not available	*cry1Ab* + *cry1Fa2* + *mcry3A* + *ecry3.Ab* + *vip3A(a)* + *pat*	Herbicide tolerance and insect resistance (lepidopteran and coleopteran)
18.	MIR162 × MON89034	Syngenta	Argentina (2016), Brazil Mexico (2017), Taiwan (2018)	4	Not available	*vip3Aa20* + *cry2Ab2* + *cry1A.105*	Lepidopteran insect resistance
19.	MIR162 × MON89034 × GA21	Monsanto Company	Brazil (2019)	1	Not available	*vip3Aa20* + *cry2Ab2* + *cry1A.105* + *mepsps*	Herbicide tolerance and lepidopteran insect resistance
20.	MIR162 × NK603	DuPont	Argentina (2016), Brazil (2015)	2	Not available	*vip3Aa20* + *cp4 epsps*	Herbicide tolerance and lepidopteran insect resistance
21.	MIR162 × TC1507	Syngenta	Argentina (2014), Brazil (2015)	2	Not available	*vip3Aa20* + *cry1Fa2* + *pat*	Herbicide tolerance and lepidopteran insect resistance
22.	MIR162 × TC1507 × GA21	Syngenta	Argentina (2014)	1	Not available	*vip3Aa20* + *cry1Fa2* + *pat* + *mepsps*	Herbicide tolerance and lepidopteran insect resistance
23.	MON810 × MIR162	DuPont	Argentina (2016), Brazil (2015)	2	Not available	*cry1Ab* + *vip3Aa20*	Lepidopteran insect resistance
24.	MON810 × MIR162 × NK603	DuPont	Argentina (2016)	1	Not available	*cry1Ab* + *vip3Aa20* + *cp4 epsps*	Herbicide tolerance and lepidopteran insect resistance
25.	MON87427 × MON89034 × MIR162 × MON87419 × NK603	Monsanto	Japan, South Korea (2018)	2	Not available	*cry1A.105* + *cry2Ab2* + *vip3Aa20* + *cp4 epsps* + *dmo* + *pat*	Herbicide tolerance and lepidopteran insect resistance
26.	MON87427 × MON89034 × MON810 × MIR162 × MON87411 × MON87419	Monsanto	Japan (2018)	1	Not available	*cry1A.105* + *cry1Ab* + *cry2Ab2* + *cry3Bb1* + *vip3Aa20* + *cp4 epsps* + *dmo* + *pat*	Herbicide tolerance and insect resistance (lepidopteran and coleopteran)
27.	MON87427 × MON89034 × MIR162 × MON87411	Monsanto	Argentina, Brazil (2018), Japan, Mexico, South Korea (2017), Taiwan (2018)	6	Not available	*cry1A.105* + *cry1Ab* + *cry2Ab2* + *cry3Bb1* + *vip3Aa20* + *dvsnf7 + cp4 epsps*	Herbicide tolerance and insect resistance (lepidopteran and coleopteran)
28.	MON87427 × MON89034 × MIR162 × NK603	Monsanto	Argentina, Brazil (2019), Colombia (2017), Japan, Mexico (2016), Philippines (2019), South Korea, Taiwan (2016)	8	Not available	*cry1A.105* + *cry2Ab2* + *vip3Aa20* + *cp4 epsps*	Herbicide tolerance and lepidopteran insect resistance
29.	MON89034 × TC1507 × NK603 × MIR162	Dow Agro Sciences LLC	Argentina (2016), Brazil Canada (2017), Colombia (2018), Mexico, Paraguay, South Korea (2017), Taiwan (2018)	8	Not available	*cry1Fa2* + *cry2Ab2* + *vip3Aa20* + *cp4 epsps*	Herbicide tolerance and lepidopteran insect resistance
30.	Bt11 × MIR162 × MON89034	Syngenta	Argentina (2016), Brazil (2017), South Korea, Taiwan (2018)	4	Not available	*cry1A.105* + *cry1Ab* + *cry2Ab2* + *vip3Aa20* + *pat*	Herbicide tolerance and lepidopteran insect resistance
31.	Bt11 × MIR162 × MON89034 × GA21	Syngenta	Argentina (2016), Brazil (2017), Mexico (2017), Philippines (2020), South Korea (2016), Taiwan (2017)	6	Not available	*cry1A.105* + *cry1Ab* + *cry2Ab2* + *vip3Aa20* + *mepsps* + *pat*	Herbicide tolerance and lepidopteran insect resistance
32.	Bt11 × MIR162 × TC1507	Syngenta	Argentina (2014)	1	Not available	*cry1Ab* + *cry1Fa2* + *vip3Aa20* + *pat*	Herbicide tolerance and lepidopteran insect resistance
33.	TC1507 × MIR162 × NK603	DuPont	Argentina (2016), Brazil, Mexico (2015)	3	Not available	*cry1F* + *vip3Aa20* + *cp4 epsps* + *pat*	Herbicide tolerance and lepidopteran insect resistance
34.	TC1507 × MON810 × MIR162	DuPont	Argentina (2016), Brazil (2015), Colombia (2016), Japan (2013), Mexico, South Korea, Taiwan (2015)	7	Not available	*cry1Ab* + *cry1Fa2* + *vip3Aa20* + *cp4 epsps* + *pat*	Herbicide tolerance and lepidopteran insect resistance
35.	TC1507 × MON810 × MIR162 × NK603	DuPont	Argentina (2016), Brazil (2015), Japan, Mexico (2013), Philippines (2015), South Korea, Taiwan (2013)	7	Not available	*cry1Ab* + *cry1Fa2* + *vip3Aa20* + *cp4 epsps* + *pat*	Herbicide tolerance and lepidopteran insect resistance

*Compiled from ISAAA GM Approval Database (http://www.isaaa.org/gmapprovaldatabase).*

The third generation *Bt* cotton contained three kinds of genes *viz*. Twin Link Plus (*cry1Ab* + *cry2Ac* + *vip3Aa19*); Bollgard 3 (*cry1Ac* + *cry2Ab* + *vip3A*); and Wide Strike 3 (*cry1Ac* + *cry1F* + *vip3A*) (Vyavhare, 2017). The Cry1Ac or Cry2Ab resistant populations of *H. armigera* showed no cross-resistance against Vip3A (Wei et al., 2017). In Ontario, Canada, Vip3A has been used in transgenic maize lines to manage *Striacosta albicosta* and Vip3A hybrids are highly recommended as these can reduce feeding injury by this pest comparative to Cry1F hybrids and non-*Bt* corn plants (Smith et al., 2017; Farhan et al., 2018). Another study confirmed the resistance development in United States field populations of *H. zea* to Cry1 and Cry2 proteins as transgenic maize expressing these genes showed ear damage (Yang et al., 2019). Recently, the effect of *Bt* cotton containing triple genes having *cry* and *vip3Aa* combinations on the life cycle of 3 populations of *H. zea* (Florida Panhandle, United States) was studied, and 100% larval mortality was obtained in all testing populations (Rabelo et al., 2020). Besides cotton and corn, the importance of *vip3* genes has been also demonstrated in transgenic cowpea plants expressing Vip3Ba proteins which were totally protected from the attack of larvae of pest *Maruca vitrata* (Bett et al., 2017). Therefore, sustainable use of Vip3A proteins in commercial *Bt* crops should be ensured which could be a potential candidate to be used in the formulation of insect pest management strategies.

## Future Perspective

There is an urgent need to explore more *vip* genes with higher insecticidal potential and use of existing Vip proteins in insect pest management strategies to control a diverse range of lepidopteran pests and insect populations resistant to transgenic crop lines expressing various Cry proteins. These toxins can be used for development of transgenic lines, biopesticide formulations, nano-emulsions, *etc*., to use them the most effectively. The focus on the exploration of novel Vip toxins and their characterization from *Bt* strains available with the scientific society or new *Bt* strains should be enhanced. These proteins should be considered as a valuable contender to be incorporated in the gene-pyramiding approach for prolonged use of respective transgenic lines and delay in the development of resistance in a particular target pest. In addition to this, it is necessary to focus on the enhancement of our understanding of receptors and signaling pathways for the effective and long-term use of these proteins.

## Author Contributions

MG and SK conceived the idea and worked on the structure, overall organization, and scope of review. MG and HK wrote the text, prepared tables, and arranged references. HK designed all figures. SK critically supervised and edited the manuscript. All authors finalized the manuscript, contributed to the article, and approved the submitted version.

## Conflict of Interest

The authors declare that the research was conducted in the absence of any commercial or financial relationships that could be construed as a potential conflict of interest.

## Footnotes

1 http://www.lifesci.sussex.ac.uk/home/Neil_Crickmore/Bt/vip.html

2 https://www.bpprc.org/

3 https://camtech-bpp.ifas.ufl.edu/categorize_database_vpa

4 https://camtech-bpp.ifas.ufl.edu/categorize_database_vpb

5 https://camtech-bpp.ifas.ufl.edu/categorize_database_vip
